# By Modulating the Hormonal Balance and Ribonuclease Activity of Tomato Plants *Bacillus subtilis* Induces Defense Response against Potato Virus X and Potato Virus Y

**DOI:** 10.3390/biom12020288

**Published:** 2022-02-10

**Authors:** Svetlana V. Veselova, Antonina V. Sorokan, Guzel F. Burkhanova, Sergey D. Rumyantsev, Ekaterina A. Cherepanova, Valentin Y. Alekseev, Elena R. Sarvarova, Albina R. Kasimova, Igor V. Maksimov

**Affiliations:** Institute of Biochemistry and Genetics, Ufa Federal Research Centre, Russian Academy of Sciences, Prospekt Oktyabrya, 71, 450054 Ufa, Russia; fourtyanns@googlemail.com (A.V.S.); guzel_mur@mail.ru (G.F.B.); rumyantsev-serg@mail.ru (S.D.R.); k_cherepanova@mail.ru (E.A.C.); valentin-1994@yandex.ru (V.Y.A.); sarvarova_lena@mail.ru (E.R.S.); albinakasimova2014@mail.ru (A.R.K.); igor.mak2011@yandex.ru (I.V.M.)

**Keywords:** plant-growth-promoting microorganisms (PGPM), endophytes, *Bacillus subtilis*, potato virus X, potato virus Y, induced systemic resistance (ISR), phytohormones, cytokinins (CK), auxins, abscisic acid (ABA)

## Abstract

Endophytic plant-growth-promoting microorganisms can protect plants against pathogens, but they have rarely been investigated as potential biocontrol agents and triggers of induced systemic resistance (ISR), regulated by phytohormones, against viruses. We studied the role of endophytic strains *Bacillus subtilis* 26D and *B. subtilis* Ttl2, which secrete ribonucleases and phytohormones, in the induction of tomato plant resistance against potato virus X and potato virus Y in a greenhouse condition. The endophytes reduced the accumulation of viruses in plants, increased the activity of plant ribonucleases and recovered the fruit yield of infected tomato plants. Both the 26D and Ttl2 strains induced ISR by activating the transcription of genes related to salicylate- and jasmonate-dependent responses. The 26D and Ttl2 strains increased the content of cytokinins and decreased the level of indolacetic acid in plants infected with PVX or PVY. PVY led to an increase of the abscisic acid (ABA) content in tomato plants, and PVX had the opposite effect. Both strains reduced the ABA content in plants infected with PVY and induced ABA accumulation in plants infected with PVX, which led to an increase in the resistance of plants. This is the first report of the protection of tomato plants against viral diseases by foliar application of endophytes.

## 1. Introduction

Plant viruses are the most dangerous plant pathogens. Since they invade all known agricultural crops, almost half of the epiphytotics in the world are of viral origin, resulting in great agronomic losses [1]. Currently, several strategies are used to protect plants against viruses: (i) the application of chemical agents with antiviral activities and pesticides to control the viral disease vector [2]; (ii) the use of plant resistance against viruses, which may depend on the plant genotype or may be induced by chemical or biological elicitors [3,4]; (iii) obtaining virus-free plants using genetic engineering methods [5] and (iv) the employment of plant-growth-promoting microorganisms (PGPM) at various levels of the plant defense against viruses [6,7].

However, the use of the first three strategies of protection is limited, since chemical antiviral compounds are toxic to plants, animals and humans, and selection for resistance to viruses and the genetic transformation of plants is costly and time-consuming [8]. Therefore, the environmentally friendly protection of plants against viruses is currently associated with the use of PGPM and their metabolites as new antiviral agents and biological elicitors [9,10,11,12]. Scientists are especially interested in endophytes, a group of bacteria and fungi that can colonize the internal tissues of plants and form the closest relations with the host plant [13,14]. Some studies have shown that endophytes are much more effective than rhizosphere and phyllosphere bacteria in protecting plants against pathogens and pests [14,15].

A large number of studies documented the activity of PGPM against viral infection, virus spread and reproduction in plants. Bacteria of the *Bacillus*, *Pseudomonas*, *Paenibacillus*, *Stenotrophomonas* and *Streptomyces* genera and beneficial fungi, such as *Fusarium* and *Trichoderma*, reduced the accumulation level of viruses in plants and induced antiviral responses against cucumber mosaic virus (CMV), tobacco mosaic virus (TMV), tomato mosaic virus (ToMV), tomato spotted wilt virus (TSWV) and tomato mottle virus (ToMoV) and against potato virus Y (PVY) and potato virus X (PVX) in tomato [10,16,17,18] and banana plants [11], pepper [8], Arabidopsis [19,20], tobacco plants [9,21,22], potato plants [23,24] and cucumber [20]. Unfortunately, there are almost no reports of the ability of these bacteria to colonize the internal tissues of plants, except for a few studies [24].

Currently, the protection of plants against pathogens, pests and viruses using PGPM is associated, first, with the various metabolites produced and showing antibiotic activity; second, with the synthesis of glucanases, proteases, ribonucleases, etc. [25,26,27]; third, with elicitor activity and triggering induced systemic resistance (ISR) [8,10,27], which occur due to bacterial microbe-associated molecular patterns (MAMPs), such as flagellin, lipopolysaccharides and others [27] and fourth, with the impact of bacteria on the RNA interference mechanism [28]. In addition, PGPM have a direct effect on plant growth, which is associated with an increase in the availability of mineral nutrition elements for plants and the production of metabolites with hormonal and signaling functions (auxins, cytokinins, gibberellins, abscisis and salicylic and jasmonic acids) [27,29]. Although the implementation of PGPM as an antiviral agent is increasingly being accepted, the mechanisms of the PGPM–host–virus interaction require further study.

Plants have a sophisticated layered immune system to battle pathogens, pests and viruses, referred to as basal systemic resistance, which, depending on the elicitors, is divided into: (i) induced systemic resistance (ISR), which is activated by PGPM, and (ii) systemic acquired resistance (SAR), which is activated by various pathogens or chemical compounds [3,30,31]. The development of both the SAR and ISR activated by PGPM leads to multiple cellular responses in plants, including an oxidative burst in the apoplast and the activation of a cascade of calcium-dependent or mitogen-activated protein kinases, which subsequently leads to the reprogramming of the transcriptome and proteome and the synthesis of pathogenesis-related proteins (PR-proteins) [30,32]. Phytohormones play a key role in the regulation of these primary immune responses upon SAR and ISR activation. Salicylic acid (SA), jasmonic acid (JA), and ethylene are the main hormones associated with the immune response of plants [31]. These phytohormones form the central regulatory network of plant immunity and interact with the plant hormones associated with growth and resistance to abiotic stressors, such as cytokinins (CK), auxins, abscisic acid (ABA), brassinosteroids (BR) and gibberellins (GA) [31,32].

PGPM and viruses can regulate the hormonal balance in plants and disrupt hormonal pathways, which in the case of PGPM leads to the initiation of ISR and in the case of viruses leads to the onset of symptoms, viral reproduction and systemic infection [10,17,27,29,31,33,34,35,36]. Thus, a comprehensive understanding of the functions performed by hormones in the pathosystems of plant viruses can contribute to the development of innovative biotechnologies and genetic and breeding approaches for improving agricultural crops [31,37].

The ability of bacteria to synthesize enzymes into the external environment, known as ribonucleases, such as binases, baRNases and baliphases, which can be used to control viruses is of great interest [38,39,40,41]. Low concentrations of ribonucleases stimulate plant growth and resistance to a broad spectrum of stress factors, and high levels of ribonucleases show antiviral properties and destroy viral RNA [25,26,42].

We have previously shown that endophytic strains of *Bacillus* spp. With a high ability to colonize the internal plant tissues, combined with high ribonuclease activity, stimulated the activity of ribonucleases in potato plants for a long time after application, reduced viral disease symptoms of potato virus M (PVM), potato virus S (PVS) and PVY on potato plants and increased potato yields under field conditions [24]. Here, we studied the effect of endophytic strains of *Bacillus* spp., which are able to synthesize cytokinins and IAA and to produce ribonucleases into the extracellular environment, on the development and distribution of PVX and PVY in tomato plants. In order to identify and quantify viruses, we employed an integrated approach using qRT-PCR and ELISA methods. We also demonstrated the positive effect of hormone-producing endophytic strains of *Bacillus* spp. on growth rates, ribonuclease activity, the induction of systemic resistance and the productivity of tomato plants infected with PVX and PVY. In addition, we provide novel evidence regarding the role of the bacteria in the regulation of the hormonal balance in plants during viral infection. These findings are useful for understanding the mechanisms underlying the manipulation of plant hormonal signaling pathways by viruses and the involvement of bacteria in the induction of hormone-regulated systemic resistance.

## 2. Materials and Methods

### 2.1. Research Objects

Bacteria: The gram-positive aerobic *B. subtilis* 26D (designated as 26D) strain from the collection of the Laboratory of Biochemistry of Plant Immunity of the Institute of Biochemistry and Genetics Ufa Federal Research Center Russian Academy of Sciences (UFRC RAS) (http://ibg.anrb.ru/wp-content/uploads/2019/04/Katalog-endofit.doc, accessed on 30 January 2022) was used. The *B. subtilis* 26D strain synthesizes the lipopeptide surfactin, exhibits proteolytic, antifungal, insecticidal and antiviral activities and also induces systemic resistance against pathogens and pests [24]. The isolate *Bacillus* sp. Ttl2 (designated as Ttl2) was collected from the wheat leaves of *Triticum timopheevii* Zhuk. grown on the territory of the Iglinsky District (54°50.48′094.0″ N; 56°26.46′09.0″ E) of the Republic of Bashkortostan (Russia). *Bacillus* sp. Ttl2 is held in the collection of the Laboratory of Biochemistry of Plant Immunity of the Institute of Biochemistry and Genetics, UFRC RAS (http://ibg.anrb.ru/wp-content/uploads/2019/04/Katalog-endofit.doc, accessed on 30 January 2022). Bacteria were grown on liquid Luria–Bertani (LB) medium (1% tryptone, 0.5% yeast extract and 0.5% NaCl) at 20–22 °C using laboratory shakers (120 rpm).

Viruses: We used the natural isolates PVX-19AA and PVY-19KV, which were obtained from single potato plants growing in the Republic of Bashkortostan. The isolate PVX-19AA was obtained from potato plants of the Agata variety growing in the Aurgazinsky District (53°58′33.6″ N 55°57′14.4″ E). The isolate PVY-19KV was collected from potato plants of the Vineta variety growing in the Kaltasinsky District (55°58′27.6″ N 54°51′29.3″ E). Plants of the Agata and Vineta varieties did not contain other viruses than isolates PVX-19AA and PVY-19KV, which was verified by DAS-ELISA (Figure S1). Viruses were maintained by propagating potato plants with tubers in an insect-proof greenhouse with an ambient temperature of 25 °C during day and 21 °C at night under natural illumination. Sap was derived from potato leaves and stored at −20 °C. The presence of viruses in the sap was checked by ELISA and the presence of a sufficient amount of viral protein was tested using a Western blot, as described below (Figure S1, see Supplementary Materials).

Plants: The objects of this study were tomato plants (*Solanum lycopersicum* L.) cultivar “Volovye Serdtse” (VS) (susceptible to viruses, according to the description of the seed-breeding company “Gavrish”) and the “Ural F1” (moderately resistant to viruses, according to the description of the seed-breeding company “Gavrish”).

### 2.2. 16S rRNA Gene Sequencing

Genomic DNA of *Bacillus* sp. Ttl2 was isolated with a lysogenic buffer that contained 1% Triton X100, 1% Tween 20, 1% Chelex 100 (BioRad Laboratories, Hercules, CA, USA) and 0.005% o-cresolsulfonephthalein. The 16S rRNA gene was amplified using the universal primers 27F (5′-CAGAGTTTGATCCTGGCT-3′) and 1492R (5′-AGGAGGTGATCCAGCCGCA-3′). Sequencing of the 16S rRNA gene fragments of the *Bacillus* sp. Ttl2 isolate was carried out using a NovaSeq 6000 Sequencing System (Illumina, San Diego, CA, USA) with NovaSeq 6000 SP Reagent Kits (Illumina, San Diego, CA, USA), and the results were deposited in the GenBank database with the accession number OK427265 (https://www.ncbi.nlm.nih.gov/nuccore/OK427265, accessed on 30 January 2022). The 16S rRNA sequence of the *Bacillus* sp. Ttl2 isolate was compared with the nucleotide sequences of other *Bacillus*-type strains in the NCBI database using the basic local alignment search tool (BLAST) algorithm (https://blast.ncbi.nlm.nih.gov/Blast.cgi, accessed on 30 January 2022). The alignment of the sequences was conducted with corresponding sequences of the available *Bacillus* species deposited in GenBank using the Clustal W, and the phylogenetic tree was inferred using the neighbor-joining method in the MegaLine software.

Isolate characteristics: the *Bacillus* sp. Ttl2 isolate was identified based on both phenotypic and biochemical characteristics [24].

### 2.3. Experimental Design

Experiments were conducted under two different sets of environmental conditions: the first in a KBW E6 plant growth chamber (Binder GmbH, Tuttlingen, Germany) and the second in an insect-proof greenhouse with controlled environmental conditions. The experiments were carried out from April to September in the greenhouse of the Institute of Biochemistry and Genetics UFRC RAS (54°46′00.4″ N 56°00′57.8″ E).

Plant growth conditions: The growth chamber conditions were set with a 16 h light photoperiod at an irradiance of 146 W/m^2^ FAR and a temperature of 25 °C/22 °C (day/night). The greenhouse conditions included a temperature typically at 25 °C/20 °C (day/night) and a photoperiod adjusted to 16 h of light with illumination supplementary to daylight provided by a JazzWay LED 12 W with a light intensity of 146 W/m^2^ FAR. The tomato plants were grown in the universal soil “Veltorf Premium” (composition: milled peat, agroperlit, limestone flour and complex mineral fertilizer) (Veltorf, Moscow, Russia) in separate pots with a volume of 1 L.

Plants of both the VC and Ural varieties were subjected to the following treatments, with 15 plants each: plants sprayed with water were used as a control (designated as Control in tables or the 0 point on graphs and histograms), plants sprayed with the *B. subtilis* 26D suspension (designated as 26D), plants sprayed with the *Bacillus* sp. Ttl2 suspension (designated as Ttl2), plants treated with *B. subtilis* 26D and after 7 days inoculated with PVX (designated as 26D + PVX) or PVY (designated as 26D + PVY), plants treated with *Bacillus* sp. Ttl2 and after 7 days inoculated with PVX (designated as Ttl2 + PVX) or PVY (designated as Ttl2 + PVY) and control plants inoculated with PVX (designated as PVX) or PVY (designated as PVY).

Bacterial treatment and virus inoculation: The 30-day-old tomato plants at the developmental stage of the fourth leaf were sprayed with 5 mL of bacterial suspension at a concentration of 10^8^ spores per mL. The control plants were sprayed with distilled water. The volumes of all solutions allowed the full moistening of leaves. After treatment for a day, the tomato plants were covered with plastic bags for better penetration of the endophytic bacteria into the plant tissues. The tomato plants were infected with sap from potato leaves affected with PVX-19AA and PVY-19KV, diluted with distilled water 1:1, on the 7th day after the bacterial treatment. The solution containing viruses was injected into the intercellular spaces of tomato leaves and the conducting bundles with a syringe into the previously made incisions [38,43]. In all cases, the vessels were transferred into darkness for 24 h.

### 2.4. Assessment of Viral Diseases

#### 2.4.1. Quantitative Real-Time Polymerase Chain Reaction (qRT-PCR) to Investigate Virus Accumulation

For viral diagnosis, test samples were selected from healthy and diseased tomato plants at 7 and 14 days after being infected with PVX and PVY and fixed in liquid nitrogen. The pool of five plants per replicate (three leaves per plant) was prepared for qRT-PCR analysis. Total RNA was extracted using TRIzol™ Reagent (Merck KGaA, Sigma-Aldrich, Darmstadt, Germany) according to the manufacturer’s instructions. The qRT-PCR was performed using specific primers for *PVX CP* (Coat Protein) and *PVY PIPO* (Pretty Interesting Potyviridae open reading frames(ORF)) genes by using the kit “Potato Virus X and Potato Virus Y” (Synthol, Moscow, Russia), according to the manufacturer’s protocols, on a CFX Connect real-time PCR Detection System device (BioRad Laboratories, Hercules, CA, USA). The housekeeping gene *SlACT* was used for the normalization of *PVX CP* and *PVY PIPO* expression in *S. lycopersicum*. Three independent biological replicates were performed for each experiment.

#### 2.4.2. Double-Antibody Sandwich Enzyme-Linked Immunosorbent Assay (DAS-ELISA)

The direct double-antibody sandwich enzyme-linked immunosorbent assay (DAS-ELISA Complete kits, Bioreba, Switzerland) was used to determine the PVX, PVY, PVM, PVS and potato leaf roll virus (PLRV) content, as described previously [24]. ELISA was performed on both challenged inoculated and non-inoculated leaves (to check systemic virus movement) of five tomato plants per replicate for each treatment at 14 dpi for the growth chamber experiments and 10 weeks after viral infection for the greenhouse experiments. In the potato plants on which the PVX-19AA and PVY-19KV isolates were maintained, ELISA was performed directly on the leaves from which the inoculum was obtained (Figure S1, see Supplementary Materials). Samples were considered positive for the presence of viruses if the absorbance value A_405_ exceeded three times a threshold value equal to the mean of the absorbance value of the healthy control samples. Three independent biological replicates were performed for each experiment.

#### 2.4.3. Western Blotting Assays

For Western blot analyses, total proteins from the leaves of the potato plants were isolated using the loading buffer (50 mM Tris-HCl, pH 8.2, 10% glycerol, 0.1% bromophenol blue, 1% b-mercaptoethanol and 2% SDS). The extract was separated through denaturing electrophoresis in 15% sodium dodecyl sulfate-polyacrylamide gel electrophoresis using a camera BioRad MiniProtean (BioRad Laboratories, Hercules, CA, USA). The protein samples were transferred to a nitrocellulose membrane “Power Blotter Select transfer packages” with a 1-stage transfer buffer Thermo Scientific Power Blotter (Thermo Fisher Scientific, Uoltem, MA, USA). Electroblotting was performed using the Power Blotter system (Thermo Fisher Scientific, Uoltem, MA, USA). The resulting membranes were blocked with an iBind Solution Kit then were placed into iBind Cards (Thermo Fisher Scientific, Uoltem, MA, USA). A flexible Western iBind Device was used for hybridization with the primary antibodies raised against *PVX CP* and *PVY CP* (Bioreba, Switzerland) (1:2000 dilution) and the secondary antibody, peroxidase-conjugated goat anti rabbit immunoglobulin G (Sigma-Aldrich, Darmstadt, Germany) (1:1000 dilution). Protein transfer and hybridization with antibodies were performed according to the manufacturer’s instructions for the devices (Thermo Fisher Scientific, Uoltem, MA, USA). The resulting blots were then visualized using 1.4 × 10^−3^ M 3,3′-Diaminobenzidine (Sigma-Aldrich, Darmstadt, Germany) and 0.02% hydrogen peroxide in 0.1 M potassium phosphate buffer with a pH of 6.8. After coloring, the membrane was rinsed with distilled water and scanned on the GelDoc Go Gel Imaging System (BioRad Laboratories, Hercules, CA, USA) (Figure S1, see Supplementary Materials).

### 2.5. Assessment of the Endophytic Properties of the Bacillus Strains

The endophytic content of the strains under investigation was evaluated by counting the colony-forming units (CFU) of microorganisms on the 5th day after the inoculation of sterile tube-grown potato plants (cv Udacha) cultivated for 25 days with a 16 h light photoperiod in the KBW E6 plant growth chamber (Binder GmbH, Tuttlingen, Germany) on the 7% agar Murashige–Skoog medium, as described earlier [24]. At least 20 plants were inoculated with 5 mL of the bacterial cell suspension (10^8^ cells/mL) of each strain (or isolate). After 5 days of co-culturing, shoots (leaves and stems) and roots of plants were surface-sterilized for 3 min in 70% ethanol. Next, they were exposed for 3 min to a 0.03% solution of hydrogen peroxide, and then the samples were washed 3 times with sterile distilled water. Sterilized plant material (40 mg fresh weight) was homogenized with 40 mL of sterile water added. Aliquots (30 μL) were dispersed over the surface of potato-glucose agar with an L-shaped spatula until complete drying. After 24 h of incubation at 28 °C in the TS-1/20 SPU chamber (Smolensk SKTB SPU, Smolensk, Russia), Petri dishes were analyzed for the count of CFU using the colony counter eCount (Heathrow Scientific, Vernon Hills, IL, USA). CFU were counted in the second and third dilutions, and their number was recalculated per 1 g of fresh plant weight.

### 2.6. Assessment of the Growth, Fresh and Dry Weight and Yield of Tomato Plants

The shoots height of each plant, from the base of the stem to the apical bud, was measured before inoculation with viruses (0 point on the graphs and histograms) and every 7 days for 5 weeks after inoculation, for 10 plants for each treatment. For each plant, the increase in shoot height was calculated after a period of 7 days (growth rate) during the 5 weeks after inoculation. The fresh weight of the tomato plants of both the VC and Ural varieties was measured 14 days after inoculation with PVX or PVY in all treatment combinations. The weighed shoots were dried in the SNOL 67/350 dry heat oven (AB Umega, Utena, Lithuania) at 75 °C until a constant weight to measure the dry weight of the shoots. The results of the fresh weight and the dry weight were recorded for five individual plants (randomly chosen from the 10 plants in each treatment) and expressed as means ± SE.

In order to measure yields, the tomato fruits from 10 plants for each treatment were harvested and weighed at 14 weeks after being infected with viruses. Tomato fruits from each plant were weighed separately and the average weight of one fruit per plant and the average weight of fruits for each treatment were calculated.

### 2.7. Assessment of Ribonuclease Activity in the Liquid Culture Medium of Bacteria and in Tomato Plants

The quantitative estimation of extracellular ribonuclease activity in the liquid culture medium was carried out according to the modified method of [44]. Culture growth was measured spectrophotometrically at 600 nm and expressed as optical density units (OD_600_). When the density of bacteria reached 10^8^ spores per mL, the cultures were centrifuged at 15,000× *g* for 15 min (5415 K Eppendorf, Hamburg, Germany). The tomato leaves (1:10 weight/volume) were homogenized in 0.05 M tris–HCl buffer (pH 8.5) and incubated for 60 min at 4 °C. The supernatants were separated by centrifugation at 15,000× *g* for 15 min (5415 K Eppendorf, Hamburg, Germany). Twenty microliters of the homogenates or centrifuged culture medium was added to 1.98 mL of Torula yeast RNA solution (50 mg/mL) (Merck KGaA, Sigma-Aldrich, Darmstadt, Germany) in 0.05 M of tris–HCl buffer (pH 8.5) and kept at 25 °C for 1 h, and its absorbance was measured at 260 nm relative to the control (mixture reaction without leaf extract or bacterial medium) at 260 nm on an LLG-uniSPEC 2 spectrophotometer (LLG, Germany). The unit of nuclease activity was taken as the amount of enzyme causing an increase in adsorption by 1.0 optical unit at 260 nm for 1 h at 25 °C [45]. Ribonuclease activity was expressed as units/(mL of liquid medium per min) (extracellular *Bacillus* ribonuclease activity) or units/(mg of protein per min) (ribonuclease activity in plants). The protein concentration in plants was measured using a Bradford assay.

### 2.8. Isolation of RNA and Performing the Quantitative Real-Time Polymerase Chain Reaction (qRT-PCR) to Investigate Defense Gene Expression of Tomato Plants

In order to study gene expressions, leaves and stems from healthy and diseased tomato plants were selected (five plants per biological replicates) at 7 and 14 days after being infected with PVX and PVY and fixed in liquid nitrogen. Total plant RNA was extracted using TRIzol™ Reagent (Merck KGaA, Sigma-Aldrich, Darmstadt, Germany) according to the manufacturer’s instructions. First strand cDNA for each sample was synthesized by using a first-strand synthesis system for quantitative real-time polymerase chain reaction (qRT-PCR) (Syntol, Moscow, Russia), following the manufacturer’s instructions. The cDNA of each sample was diluted to 200 ng/μL before amplification. Primers for qRT-PCR were designed using a web-based primer designing tool from IDT (http://eu.idtdna.com/Scitools/Applications/Primerquest, accessed on 30 January 2022). The sequences of all the primers are presented in Table S1 (Supplementary Materials). The mRNA expression levels of the selected probes were analyzed by qRT-PCR using Eva-Green Supermix (Syntol, Moscow, Russia), according to the manufacturer’s protocols, on a CFX-Connect thermo-cycler (BioRad Laboratories, Hercules, CA, USA) with Bio-Rad CFX Maestro Optical System Software (BioRad Laboratories, Hercules, CA, USA). For the calculation of the threshold cycle (CT) values, the auto-CT function was used. Each sample was analyzed in three technical replicates, and for further calculations the mean value of each triplicate was used. To normalize the target gene expression, the difference between the CT of the target gene and the CT of Actin (constitutive control) (Table S1, see Supplementary Materials) for the respective template was calculated (ΔCT value). To calculate fold changes (FC) in gene expression, the ΔCT value was calculated as follows: ΔCT = CT (target gene) − CT (constitutive control gene). The relative transcript levels were calculated as: 2^−ΔCT^. Three independent biological replicates were performed for each experiment.

### 2.9. Assessment of the Phytohormone Content in the Liquid Culture Medium of Bacteria and in Tomato Plants

Leaves and stems from five tomato plants (approximately 1 g) per one biological replication on the 7th and 14th days after inoculation with viruses were homogenized and phytohormones (cytokinins, indoleacetic acid (IAA) and abscisic acid (ABA)) were extracted with 80% ethanol (1:10, weight/volume) for 16 h at 4 °C. The extract was separated by 20 min of centrifugation at 4000× *g* in an Avanti J-E centrifuge (Bekman Coulter, Bray, CA, USA) and evaporated to obtain aqueous residue. The liquid culture medium obtained by cultivating *B. subtilis* 26D and *B. subtilis* Ttl2 was collected at the late logarithmic growth phase or at the beginning of the stationary phase (on the third day) and centrifuged at 4000× *g* for 20 min in an Avanti J-E centrifuge (Beckman Coulter, Bray, OK, USA). The supernatant was analyzed for the content of phytohormones (cytokinins, IAA and ABA). Three independent biological replicates were performed for each experiment.

#### 2.9.1. Cytokinins Assay

Cytokinins from 2 mL of the supernatant of the bacterial culture liquid were twice extracted with n-butyl alcohol in a 2:1 ratio (aqueous phase/organic phase). The extract was evaporated to dryness. Cytokinins from the aqueous residue of the plant extract were concentrated on a C18 column (Waters Corporation, Milford, MA, USA), eluted with 5 mL of 80% ethanol and then evaporated to dryness. Cytokinin bases and their derivatives from the dry residue in both cases were separated by thin layer chromatography on silufol plates (Merck KGaA, Fluka, Darmstadt, Germany) in the system of solvents, butanol: ammonium hydrate: water (6:1:2), according to the work of [46]. In this work, we analyzed the riboside of zeatin (ZR, Rf 0.4–0.5) and zeatin (Z, Rf 0.6–0.7). The material from different zones was eluted with 0.1 M PB, pH 7.4 for 16 h. Then, the silica gel was removed by 10 min of centrifugation at 10,000× *g* in an Eppendorf 5415 K centrifuge. In the supernatant, phytohormone was assayed by means of ELISA using specific antibodies, as described earlier [46]. Anti-trans-ZR sera were used for the assay of the cytokinins of Z types [46]. Their specificity has been described previously [46,47].

#### 2.9.2. IAA and ABA Assays

IAA and ABA from 1 mL of the supernatant of the bacterial culture liquid and from an aliquot of the aqueous residue of plant material were extracted with diethyl ether, according to a modified scheme [48]. The extraction was carried out from the aqueous residue, which was acidified with HCl, with diethyl ether, followed by extraction into a sodium bicarbonate solution and re-extraction into diethyl ether (after acidification of sodium bicarbonate) with a decrease in volume at each stage of extraction-re-extraction, after which the samples were methylated with diazomethane [49]. An IAA and ABA quantitative assay was performed with ELISA using specific antibodies, as described previously [49]. The reliability of the phytohormone immunoassay was confirmed using a dilution test and through a comparison with the data obtained with the results of high performance liquid chromatography (HPLC) in combination with mass spectrometry [46,50].

### 2.10. Statistics

The experiments were performed two times with three replicates for each treatment (five plants per replicate), except for the measurements of shoots height, the fresh and dry weights of shoots and fruits yields, which were performed in 10, 5 and 10 biological replications, respectively, for each treatment. The assessment of the endophytic properties was carried out in 20 biological replicates. The experimental data were expressed as means ± SE, which were calculated in all treatments using MS Excel. An analysis of variance (ANOVA) was used to calculate the least significance difference (LSD) at *p* ≤ 0.05 to discriminate means.

## 3. Results

### 3.1. Characterization of the B. subtilis 26D and B. subtilis Ttl2 Strains

In our work, the *B. subtilis* 26D strain was used, which is the basis of the biological control agent “Phytosporin”. The properties of this strain were described earlier [24,51].

**Figure 1:**
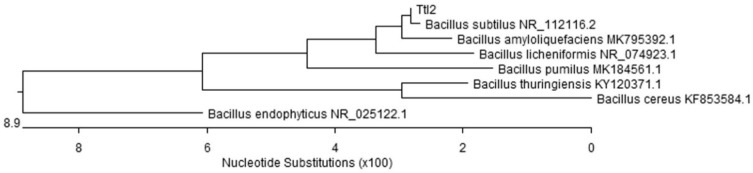
Phylogenetic tree based on the 16S rRNA gene sequences from *Bacillus subtilis* Ttl2 and other *Bacillus* spp.

The physiological, morphological and biochemical properties of the *Bacillus* sp. Ttl2 isolate are represented in Supplementary Table S2. Sequencing of the 16S rRNA gene from *Bacillus* sp. Ttl2 isolate was performed and compared with other nucleotide sequences of *Bacillus*-type strains. A phylogenetic tree was generated using the neighbor-joining method (Figure 1). The *Bacillus* sp. Ttl2 isolate exhibited a sequence similarity (99.9%) with the *B. subtilis* strain and was designated as *Bacillus subtilis* Ttl2 (Figure S2, see Supplementary Materials).

Both bacteria were endophytic (Table 1). The number of *B. subtilis* 26D colony forming units (CFU) in the tissues of plant shoots after surface sterilization was approximately 13.1 × 10^4^ per g of plant fresh weight, and the number of *Bacillus* sp. Ttl2 was half the amount of living *B. subtilis* 26D cells (Table 1). The content of living *B. subtilis* 26D cells in the plant roots was substantially lower than in the shoots and achieved 0.17 × 10^4^ CFU per g of plant fresh weight. We found about 1.0 × 10^4^ CFU of *B. subtilis* Ttl2 per g of fresh weight of roots.

Both bacteria exhibited ribonuclease activity. Significant ribonuclease activity was observed in the liquid culture medium of *B. subtilis* 26D (Table 1). Interestingly, the ribonuclease activity in the *B. subtilis*. Ttl2 suspension was 1.7 times less than that in *B. subtilis* 26D culture medium (Table 1). Both bacteria produced hormones into the culture medium: cytokinins and auxins but not ABA (Table 1). *B. subtilis* 26D produced more cytokinins (the sum of zeatin and zeatin riboside), and *B. subtilis* Ttl2 secreted 2 times more auxins (IAA) than *B. subtilis* 26D (Table 1).

### 3.2. Endophytic Strains B. subtilis 26D and B. subtilis Ttl2 Suppress the Accumulation of PVX and PVY in Tomato Plants

**Figure 2:**
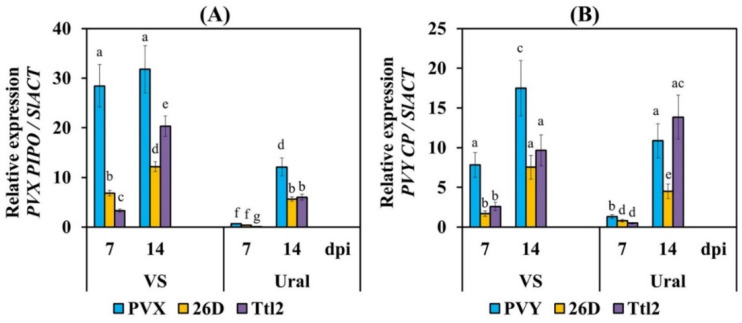
Influence of endophytic strains *B. subtilis* 26D and *B. subtilis* Ttl2 on the expression of the viral genes *PVX CP* (**A**) and *PVY PIPO* (**B**) in tomato plants of varieties Volovye Serdtse (VS) and Ural at 7 and 14 days post-inoculation (dpi). The expression values were normalized to the housekeeping gene of tomato plants *SlACT* as an internal reference. The samples are indicated as follows: PVX, plants infected with potato virus X; PVY, plants infected with potato virus Y; 26D, plants treated with *B. subtilis* 26D; Ttl2, plants treated with *B. subtilis* Ttl2. The figures present means ± SE (n = 6). The treatments marked with similar Latin letters in one cultivar do not differ significantly, according to the LSD test (*p* ≤ 0.05).

In order to test the effect of the bacterial strains on the accumulation and spread of PVX and PVY in tomato plants, we studied the expression of the *PVX CP* and *PVY PIPO* viral genes at 7 and 14 days post-inoculation (dpi) and determined the presence of the viruses using DAS-ELISA 2 and 10 weeks after infection (Figure 2, Table S3).

The expression of viral genes was quantified using qRT-PCR based on the ratio of the expression of the *PVX CP* and *PVY PIPO* genes and the housekeeping gene of tomato plants, *SlACT*. An analysis of the transcriptional activity of the *PVX CP* viral gene in plants showed that already on the 7th day after virus infection, RNA abundance was very high in the leaves of cv. VS, and a small amplification of *PVX CP* was detected in the leaves of cv. Ural (Figure 2). The transcript levels of the *PVX CP* gene remained similarly high in plants of cv. VS and were 2.6 times lower in plants of cv. Ural on the 14th dpi (Figure 2). Treatment of *B. subtilis* 26D and *B. subtilis* Ttl2 reduced virus RNA accumulation in both varieties by 2 to 6 times compared to the untreated infected plants during 2 weeks of infection (Figure 2).

An analysis of the transcriptional activity of the *PVY PIPO* viral gene in the plants showed that the level of viral RNA amplification in cultivar VS was 6 times higher than that in cultivar Ural after 7 dpi (Figure 2). A significant transcript accumulation of the *PVY PIPO* gene was detected in plants of both cultivars at 14 dpi, but the transcript levels of the *PVY PIPO* gene in Ural plants were 2 times lower than those in VS plants (Figure 2).

Treatment with bacteria reduced the viral RNA accumulation in plants of both cultivars at 7 and 14 dpi by approximately 1.8 to 4.7 times, but the transcript levels of the *PVY PIPO* gene did not decrease in the Ttl2 + PVY treatment in Ural plants (Figure 2).

The results obtained by PCR were further confirmed by ELISA (Table S3). Moreover, the ELISA analysis also showed a decrease in the PVX and PVY titers in the leaves of tomato plants treated with *B. subtilis* 26D and *B. subtilis* Ttl2 during flowering and early fruiting (10 weeks after infection) (Table S3). The lowest titer of viral particles of both viruses under investigation was achieved in cv. Ural plants treated with *B. subtilis* 26D (Table S3).

### 3.3. Endophytic Strains B. subtilis 26D and B. subtilis Ttl2 Promote Plant Growth, Biomass Accumulation and Fruit Yield of Tomato Plants Infected with PVX and PVY

**Figure 3:**
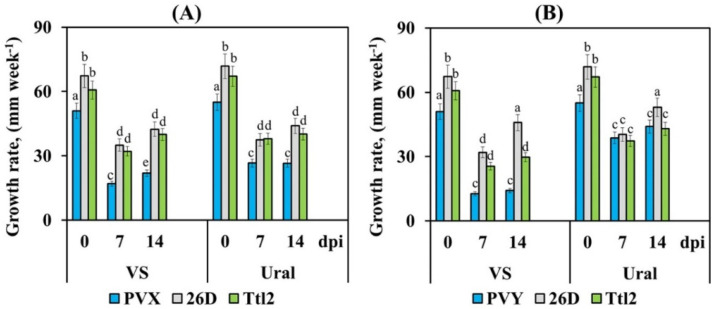
Influence of endophytic strains *B. subtilis* 26D and *B. subtilis* Ttl2 on the growth rate in tomato plants of varieties Volovye Serdtse (VS) and Ural infected with PVX (**A**) and PVY (**B**). The samples are indicated as follows: 0 dpi, control non-infected plants; PVX, plants infected with potato virus X; PVY, plants infected with potato virus Y; 26D, plants treated with *B. subtilis* 26D; Ttl2, plants treated with *B. subtilis* Ttl2. The figures present means ± SE (n = 6). The treatments marked with different Latin letters in one cultivar are statistically different from the control ones (0 dpi, not infected with viruses and not treated with bacteria) in each time-point, according to the LSD test (*p* ≤ 0.05).

We found that the growth rate of PVX-infected plants was reduced by approximately 3 times (by 57–67%) in VS plants and 2 times (by 52%) in Ural plants at the early stage of infection (1–2 weeks) (Figure 3). Later (3–5 weeks after infection), the growth rate of the PVX-infected plants of both varieties did not recover (Figure S3, see Supplementary Materials). The growth rate of the PVY-infected plants was reduced by 4 times (72–75%) in the VS plants and by 2–1.4 times (15–30%) in the Ural plants at the early stage of infection (1–2 weeks) (Figure 3). Subsequently (3–5 weeks after infection), the growth rate of the VC plants infected with PVY did not recover. The growth rate of the Ural plants infected with PVY recovered to control values after 3 weeks of infection (Figure S3, see Supplementary Materials).

Treatment with endophytic bacteria increased the growth rate of the uninfected plants of both varieties (Figure S3, see Supplementary Materials). Treatment with strains 26D and Ttl2 reduced the effects of viruses and increased the growth rate in the plants infected with PVX and PVY. The growth rate of the VC plants was restored by 30–40% in the treatments 26D + PVX and Ttl2 + PVX and was recovered by only 20–32% in the Ttl2 + PVY treatment in comparison with the infected non-bacterized plants after 1–2 weeks of infection (Figure 3).

The growth rate of the Ural plants was restored by 20–32% in the treatments 26D + PVX and Ttl2 + PVX after 1–2 weeks of infection in comparison with the infected non-bacterized plants (Figure 3). The growth rate decreased by just 10–30% in all the treatments of PVY, 26D + PVY and Ttl2 + PVY compared with the uninfected control plants (Figure 3). The growth rate was restored to control values in both varieties in the 26D + PVY treatment at 2 weeks post-inoculation (Figure 3). The growth rate of the plants containing endophytes was restored to control values in both varieties in all treatments in 3–4 weeks after infection with PVX and PVY except for the VS plants with the Ttl2 + PVY treatment (Figure S3).

A reduction of the growth rate of the infected tomato plants of both varieties under investigation led to a decrease in plant height and less accumulation of fresh and dry biomass (Table 2, Figure S3). PVX and PVY had a much stronger effect on these indicators in the plants of cv. VS (Table 2). Treatment with 26D and Ttl2 restored plant height and biomass accumulation in the PVX- and PVY-infected plants of both varieties to the level of the uninfected control plants in all treatments except for the Ttl2 + PVY treatment in VS (Table 2, Figure S3).

Subsequently, the infection with viruses led either to a complete loss of yield in the treatments VC + PVX, VC + PVY and Ural + PVX or to a decrease in the yield by 70% in the treatment Ural + PVY (Table 3). When treated with 26D and Ttl2, the tomato plants of both varieties infected with PVX or PVY restored yields by 30–105% in comparison to the uninfected and untreated control (Table 3). Strain 26D had the greatest impact on the recovery of yields of the Ural plants infected with PVY and PVX, where the yields were 105% and 80.1%, respectively, compared to the uninfected and untreated control (Table 3). Treatment with 26D of PVY-infected plants of cv. VS restored the yield to 52.7% compared to the uninfected and untreated control (Table 3). The Ttl2 strain restored the yield of the tomato plants of both varieties infected with viruses by only 34–58% compared to the uninfected and untreated control (Table 3).

### 3.4. Endophytic Strains B. subtilis 26D and B. subtilis Ttl2 Increase Ribonuclease and Transcriptional Activity of the Pathogenesis-Related Genes PR4 and PR10 in Tomato Plants Infected with PVX and PVY

**Figure 4:**
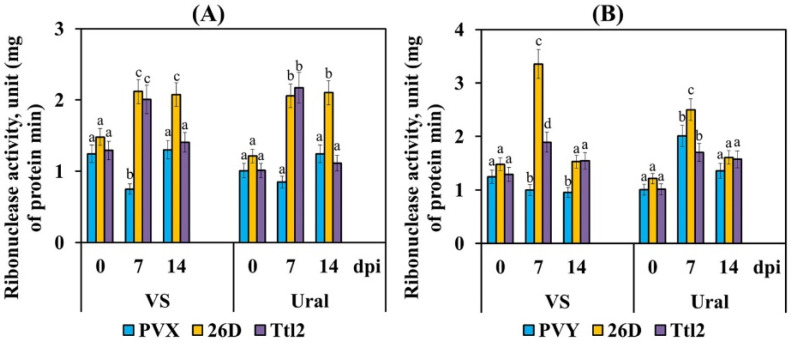
Influence of the endophytic strains *B. subtilis* 26D and *B. subtilis* Ttl2 on the ribonuclease activity in tomato plants of the varieties Volovye Serdtse (VS) and Ural infected with PVX (**A**) and PVY (**B**). The samples are indicated as follows: 0 dpi, control non-infected plants; PVX, plants infected with potato virus X; PVY, plants infected with potato virus Y; 26D, plants treated with *B. subtilis* 26D; Ttl2, plants treated with *B. subtilis* Ttl2. The figures present means ± SE (n = 6). The treatments marked with different Latin letters in one cultivar are statistically different from the control ones (0 dpi, not infected with viruses and not treated with bacteria) in each time-point, according to the LSD test (*p* ≤ 0.05).

The ribonuclease activity decreased only in the plants of cv. VS infected with PVX or PVY, while the ribonuclease activity did not change in the plants of cv. Ural infected with PVX and increased in the Ural plants infected with PVY (Figure 4). Treatment with strains 26D and Ttl2 increased the ribonuclease activity in both varieties infected with PVX and PVY (Figure 4). However, strain 26D affected this parameter much more strongly than strain Ttl2 (Figure 4).

**Figure 5:**
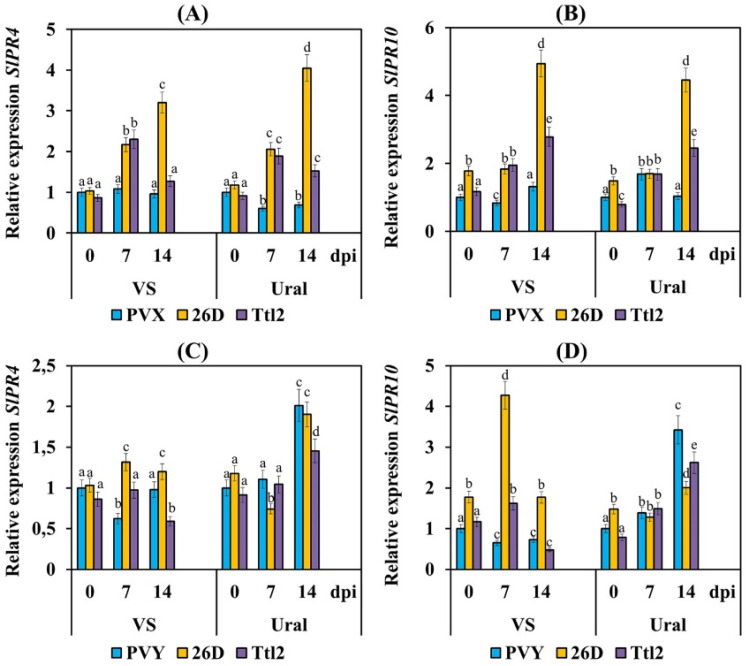
Influence of endophytic strains *B. subtilis* 26D and *B. subtilis* Ttl2 on the relative expression of the genes *SlPR4* (**A**,**C**) and *SlPR10* (**B**,**D**) in tomato plants of the varieties Volovye Serdtse (VS) and Ural infected with PVX (**A**,**B**) and PVY (**C**,**D**). The expression values were normalized to the housekeeping gene of tomato plants, *SlACT*, as an internal reference. The samples are indicated as follows: 0 dpi, control non-infected plants; PVX, plants infected with potato virus X; PVY, plants infected with potato virus Y; 26D, plants treated with *B. subtilis* 26D; Ttl2, plants treated with *B. subtilis* Ttl2. The figures present means ± SE (n = 6). The treatments marked with different Latin letters in one cultivar are statistically different from the control ones (0 dpi, not infected with viruses and not treated with bacteria) in each time-point, according to the LSD test (*p* ≤ 0.05).

It is known that members of two families of PR proteins (PR4 and PR10) in some cases have nuclease activity [52]. Inoculation of the plants of both varieties with PVX resulted in either an absence of accumulation or in a decrease in the transcript levels of the *PR4* and *PR10* genes (Figure 5). Inoculation with PVY led to a decrease in the mRNA content of the *PR4* and *PR10* genes only in the plants of cv. VS (Figure 5). Increased transcript levels of the *PR4* and *PR10* genes were found in the Ural plants infected with PVY (Figure 5).

Infection with PVX and PVY of plants of both varieties, which were treated with 26D and Ttl2, increased the transcript levels of the *PR4* and *PR10* genes in all treatments except Ttl2 + PVY in the VS plants (Figure 5). At the same time, the Ttl2 strain had a weaker effect on the transcription of these genes in both varieties than that of strain 26D (Figure 5).

### 3.5. Endophytic Strains B. subtilis 26D and B. subtilis Ttl2 Induce Systemic Resistance in Tomato Plants Infected with PVX and PVY

We analyzed the expression of the SA-dependent *SlPR1b.1* and *SlPR5* genes and the expression of the genes related to the JA-dependent response (*SlPR6* and *SlLOX*) in tomato plants infected with PVX and PVY.

**Figure 6:**
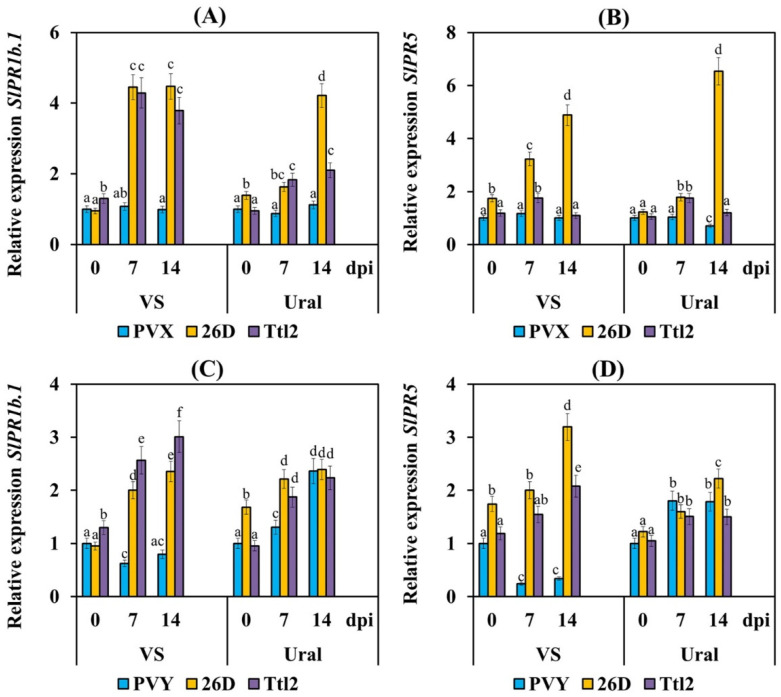
Influence of the endophytic strains *B. subtilis* 26D and *B. subtilis* Ttl2 on the relative expression of the SA-dependent genes *SlPR1b.1* (**A**,**C**) and *SlPR5* (**B**,**D**) in tomato plants of the varieties Volovye Serdtse (VS) and Ural infected with PVX (**A**,**B**) and PVY (**C**,**D**). The expression values were normalized to the housekeeping gene of tomato plants, *SlACT,* as an internal reference. The samples are indicated as follows: 0 dpi, control non-infected plants; PVX, plants infected with potato virus X; PVY, plants infected with potato virus Y; 26D, plants treated with *B. subtilis* 26D; Ttl2, plants treated with *B. subtilis* Ttl2. The figures present means ± SE (n = 6). The treatments marked with different Latin letters in one cultivar are statistically different from the control ones (0 dpi, not infected with viruses and not treated with bacteria) in each time-point, according to the LSD test (*p* ≤ 0.05).

The transcript levels of the *SlPR1b.1* and *SlPR5* genes either did not change or decreased in both varieties infected with PVX, and it significantly decreased in the plants of cv. VS infected with PVY (Figure 6). An accumulation of mRNA of the *SlPR1b.1* and *SlPR5* genes was found in the Ural plants infected with PVY (Figure 6).

Treatment with strains 26D and Ttl2 increased the transcript levels of the *SlPR1b.1* and *SlPR5* genes in both cultivars infected with both PVX and PVY, in particular in the susceptible VS plants (4 fold in the PVX-infected plants and 2 fold in the PVY-infected plants in relation to non-treated and non-infected ones) (Figure 6). Treatment with strain 26D led to an increase of the mRNA abundance of the *SlPR5* gene in the PVX-infected and PVY-infected plants of both varieties. Ttl2 showed this impact only in the case of PVY infection (Figure 6), but mRNA accumulation of the *SlPR5* gene in the plants treated with Ttl2 was 1.5 fold less than in the 26D-treated ones (Figure 6).

**Figure 7:**
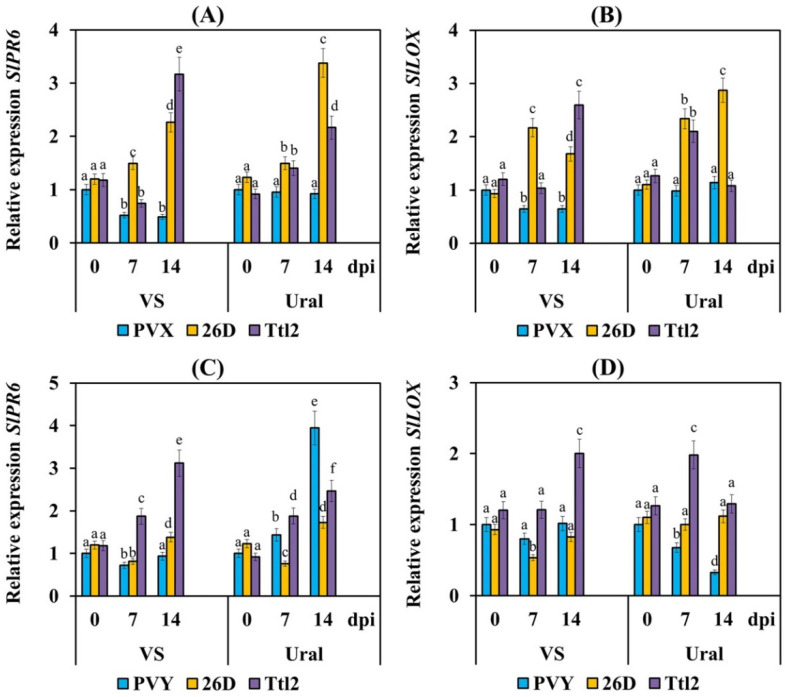
Influence of the endophytic strains *B. subtilis* 26D and *B. subtilis* Ttl2 on the relative expression of the JA-dependent genes *SlPR6* (**A**,**C**) and *SlLOX* (**B**,**D**) in tomato plants of the varieties Volovye Serdtse (VS) and Ural infected with PVX (**A**,**B**) and PVY (**C**,**D**). The expression values were normalized to the housekeeping gene of tomato plants, *SlACT,* as an internal reference. The samples are indicated as follows: 0 dpi, control non-infected plants; PVX, plants infected with potato virus X; PVY, plants infected with potato virus Y; 26D, plants treated with *B. subtilis* 26D; Ttl2, plants treated with *B. subtilis* Ttl2. The figures present means ± SE (n = 6). The treatments marked with different Latin letters in one cultivar are statistically different from the control ones (0 dpi, not infected with viruses and not treated with bacteria) in each time-point, according to the LSD test (*p* ≤ 0.05).

The transcript levels of the JA-dependent genes *SlPR6* and *SlLOX* decreased in the VS plants and did not change in the Ural plants infected with PVX (Figure 7). PVY did not influence the mRNA accumulation of the *SlPR6* and *SlLOX* genes in the susceptible VS plants but increased the transcript levels of *SlPR6* and decreased the transcript levels of the *SlLOX* gene in the resistant Ural plants. Treatment with strains 26D and Ttl2 increased the mRNA accumulation of the *SlPR6* and *SlLOX* genes in both varieties infected with PVX (Figure 7), and the impact of Ttl2 on the VS plants was more noticeable than 26D. Treatment with 26D led to a slight decrease in the transcript levels of the *SlPR6* gene in the tomato plants of cv. VS and a decrease in the mRNA accumulation of this gene in the Ural plants infected with PVY (Figure 7). The transcriptional activity of the *SlLOX* gene was not affected by the 26D treatment in both varieties infected with PVY. Treatment with Ttl2 led to an increase by 1.2–2 times of the transcript levels of the *SlLOX* gene in both varieties infected with PVY (Figure 7).

### 3.6. Endophytic Strains B. subtilis 26D and B. subtilis Ttl2 Regulate the Level of Phytohormones in Tomato Plants Infected with PVX and PVY

**Figure 8:**
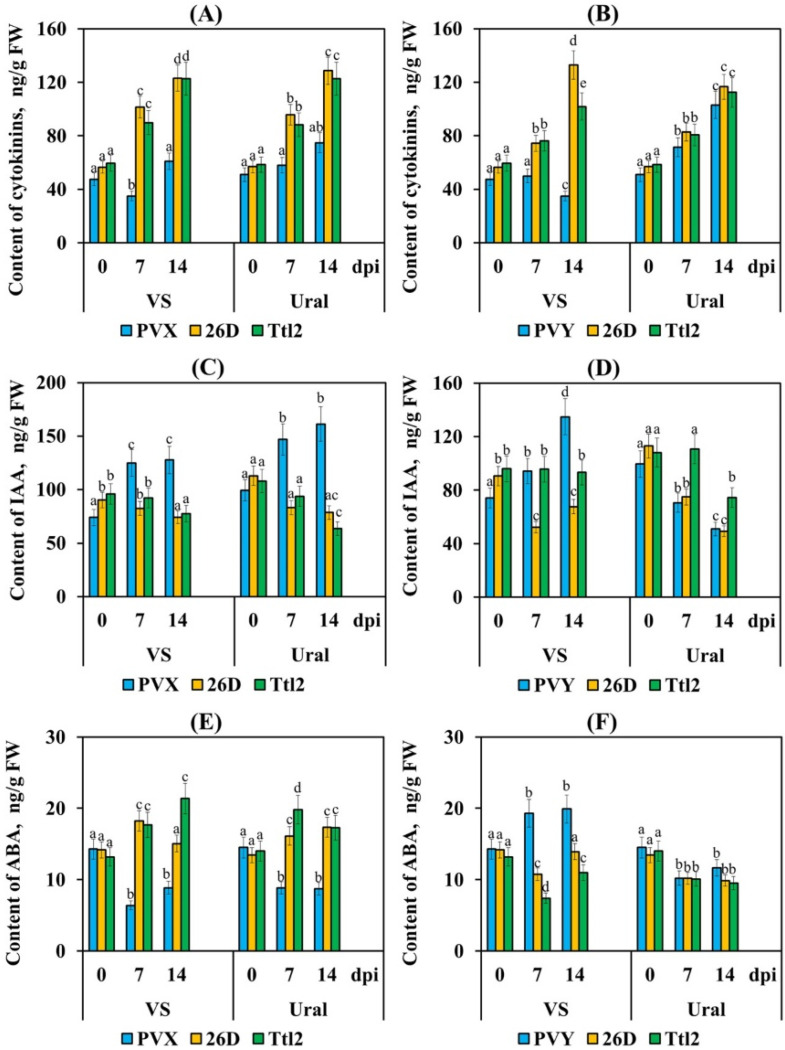
Influence of the endophytic strains *B. subtilis* 26D and *B. subtilis* Ttl2 on the content of active cytokinins (zeatin + zeatin riboside) (**A**,**B**), IAA (**C**,**D**) and ABA (**E**,**F**) in tomato plants of the varieties Volovye Serdtse (VS) and Ural infected with PVX (**A**,**C**,**E**) and PVY (**B**,**D**,**F**). The samples are indicated as follows: 0 dpi, control non-infected plants; PVX, plants infected with potato virus X; PVY, plants infected with potato virus Y; 26D, plants treated with *B. subtilis* 26D; Ttl2, plants treated with *B. subtilis* Ttl2. The figures present means ± SE (n = 6). The treatments marked with different Latin letters in one cultivar are statistically different from the control ones (0 dpi, not infected with viruses and not treated with bacteria) in each time-point, according to the LSD test (*p* ≤ 0.05).

An analysis of the hormonal balance in the tomato plants using ELISA showed that the content of active CK (zeatin + zeatin riboside) decreased or did not change, the concentration of IAA increased by 1.5–1.7 times and content of ABA decreased by 1.6–2.2 times in both varieties infected with PVX at 7 and 14 dpi (Figure 8). The content of CK increased by 1.9–2.6 times, the concentration of IAA decreased to control values and the ABA content increased by about 1.4–1.5 times in the 26D- and Ttl2-treated plants of both varieties infected with PVX at 7 and 14 dpi (Figure 8). In the plants of cv. VS infected with PVY, the content of CK decreased at 14 dpi and the concentrations of IAA and ABA increased by about 1.8 and 1.4 times, respectively, at 7 and 14 dpi (Figure 8). The CK content increased by 1.4–2 times, while the content of IAA and ABA decreased by 2 and 1.4 times, respectively, in the Ural plants infected with PVY at 7 and 14 dpi (Figure 8). The content of CK increased by 1.4–2.8 times in the bacterial-treated PVY-infected plants of both varieties (Figure 8). The ABA content decreased by 1.3–1.9 times in bacterial-treated PVY-infected plants of both varieties (Figure 8). Treatment with bacterial strains had different effects on the auxin content in the PVY-infected plants. Treatment with 26D led to a 1.4-fold decrease in the IAA content in cv. VS and a 2-fold decrease in cv. Ural (Figure 8). On the contrary, treatment with Ttl2 led to an increase in the IAA content compared to the control values in the Ural plants and by 1.3 times in the VS plants (Figure 8).

## 4. Discussion

### 4.1. Effect of Potato Virus X and Potato Virus Y on Tomato Plants

In this research we have studied the two most economically important viruses, potato virus X (PVX) and potato virus Y (PVY). PVX is a member of the genus *Potexvirus*, family *Alphaflexiviridae*, which is a worldwide group of economically important single-stranded positive-sense RNA viruses [53]. Potato virus X can infect a wide range of major crops in the *Solanaceae* family, including tomato, potato, pepper and tobacco. PVX proteins, such as the triple-gene-block (TGB) virus movement proteins (MPs), in particular P25, perform multiple functions during infections [54,55]. The silencing suppressor P25 with nucleotide binding and RNA helicase activity can modify plasmodesmata to support in the movement of the virus from cell to cell, and reorganizes the actin to contribute to PVX replication [56,57,58,59].

PVY is the most economically important virus that infects not only potato but also other species of the *Solanaceae* family, such as tomato, pepper, and tobacco, and is transmitted by 65 different aphid species in a nonpersistent manner [4]. PVY is a typical member of the genus *Potyvirus*, family *Potyviridae,* which is the largest group of RNA viruses in plants. The most studied PVY proteins are the helper component proteinase (HCPro) and the highly conserved polypeptide termed PIPO (Pretty Interesting Potyviridae ORF) [60]. Viral proteins have several functions in the life cycle of a potyvirus. For example, HCPro plays a role in aphid transmission, viral movement, pathogenicity determination and the suppression of host gene silencing [4].

Despite the fact that PVX and PVY are well studied, the effect of PGPM on them has been weakly studied. Thus, some studies have shown the induction of systemic resistance under the influence of *Bacillus amyloliquefaciens* MBI600 in tomato plants and *B. vallismortis* EXTN-1 in potato plants infected with PVX and PVY [10,23]. The antiviral activity of *Bacillus* spp. strains in potato plants against PVY and *B. pumilus* 7P/3–19 in pea and tobacco plants against PVX was also shown [24,38].

Tomato (*Solanum lycopersicum* L.) is the most important vegetable crop in the world and 136 viral species have been described that infect tomato crops. Among them are members of the genera *Begomovirus*, *Potexvirus*, *Potivirus* and others [61,62]. The most important viruses infecting tomato plants are tomato yellow leaf curl virus (TYLCV), tomato mosaic virus (ToMV) and tomato spotted wilt virus (TSWV), and tomato plants are also affected by cucumber mosaic virus (CMV), tobacco mosaic virus (TMV), potato virus Y (PVY) and potato virus X (PVX) [8,10,61,62].

Our results showed that the VS variety was susceptible to both PVX and PVY. The Ural variety showed medium susceptibility to PVX and medium resistance to PVY. According to the qRT-PCR and ELISA results, the accumulation of PVX and PVY was much higher in the VS variety than in the Ural variety (Figure 2, Table 2). In addition, viruses reduce the growth rate of VS plants much more strongly than of the Ural variety (Figure 3). The fruit yield of the VS variety plants was completely lost when they were affected by each virus. In the Ural variety, the fruit yield was absent upon PVX infection and decreased by 70% upon PVY infection (Table 3).

### 4.2. Antiviral Activity of the Endophytic Strains B. subtilis 26D and B. subtilis Ttl2

The endophytes are a broad-spectrum group of PGPMs that are able to colonize internal plant tissues and live asymptomatically inside their host. Some of them play a crucial role in the growth, development and adaptation of plants to environmental conditions [14]. Endophytic bacteria can have a number of economically useful properties: antagonism to pathogens and/or insecticidal activity, the ability to mobilize and/or fix elements of mineral nutrition of plants (phosphorus and nitrogen), degradation of toxins, synthesis of various enzymes and hormones, protection against abiotic and biotic stresses and the induction of immunity in plants [14,27]. PGPM have been used for years as environmentally friendly biocontrol agents against pathogens and pests [14,27], and only recently they have begun to be used against plant viruses [7].

In this study, we investigated the different properties of two endophytic strains, *B. subtilis* 26D and *B. subtilis* Ttl2. Our data showed that treatment with 26D and Ttl2 significantly reduced the content of both PVX and PVY in tomato plants of the VS and Ural varieties, both at 14 dpi under plant growth chamber conditions and at the stage of flowering and early fruiting (10 weeks after infection) (Figure 2, Table 2). However, the 26D strain reduced the viral titer much more strongly than the Ttl2 strain under greenhouse conditions, probably because the number of *B. subtilis* Ttl2 was half the amount of living *B. subtilis* 26D cells (Table 2). The ability to reduce viral spreading in the early stages of infection (7–14 dpi) was shown by ELISA with the *B. amyloliquefaciens* strain MBI600 on tomato plants infected with PVY [10] and with *Streptomyces ovatisporus* LC597360 on tomato plants infected with ToMV [22]. PGPM treatment also reduced the virus titer in plants when grown in greenhouse or field conditions [8,10,24]. Three-year field tests revealed a consistently reduced accumulation of CMV, broad bean wilt virus (BBWV) and Pepper mild mottle virus (PepMoV) in *B. amyloliquefaciens* 5B6-treated pepper plants [8]. In order to protect plants against viruses, aerial plant parts were sprayed with the suspension 5B6 strain, which has been shown to be an efficient technique, even against viruses in the field [8]. In our experiments, we also used the technique of a one-time spraying of plants, which had been confirmed to be effective. Thus, both bacterial strains *B. subtilis* 26D and *B. subtilis* Ttl2 showed antiviral activity, both under plant growth chamber conditions and under greenhouse conditions. However, the effect of *B. subtilis* 26D was stronger, especially when applied to the medium-resistant Ural variety.

### 4.3. The Hormone-Producing Endophytic Strains B. subtilis 26D and B. subtilis Ttl2 Increase the Growth and Fruit Yield of Tomato Plants Infected with PVX and PVY

Viral infection can cause serious inhibition of plant growth and loss of yield. We found a positive effect of bacterial treatment on the growth rate of plants infected with viruses (Figure 3 and Figure S3). Already, after 14 dpi, the growth rate was restored by 30–60% in the bacteria-treated plants, and 4 weeks after infection, the growth rate in bacterized plants was restored by 100% and reached the control values. On the other hand, plants of both varieties untreated with bacteria and infected with viruses could not fully restore the growth rate, with the exception of the PVY-infected Ural variety (Figure 3 and Figure S3). Our results showed that treatment with 26D and Ttl2 bacteria also had a positive effect on the accumulation of fresh and dry biomass. All this led to a recovery of the fruit yield by 30–105% compared to the control in both varieties infected with PVX and PVY (Table 3). The *B. subtilis* 26D strain had the greatest effect on the growth and productivity of tomato plants infected with viruses. Many reports showed that the application of some *Bacillus* spp., *Pseudomonas* spp. and *Streptomyces* spp., improved plant growth, the tuber and fruit numbers and weight, as well as the fresh and dry weight compared with the untreated infected plants and significantly reduced the negative effects resulting from the viral infection [8,9,10,12,22,24].

In recent years, the mechanisms of increasing plant growth by PGPM, including endophytic bacteria, have been studied by many researchers. Bacteria can influence plant growth by producing phytohormones, such as indole acetic acid [63,64,65,66], cytokinins and gibberellins [47,65,66,67]. There is a lot of information in the literature on the ability of endophytic microorganisms to synthesize IAA. About 34% of endophytes possess the ability to synthesize auxin IAA [14]. There is much less information about cytokinin-producing bacteria, and it is mainly associated with rhizosphere microorganisms [65]. Thus, the rhizosphere strain *B. subtilis* IB-22 produced zeatin-riboside, and in lettuce plants inoculated with this strain, the growth and weight of roots increased by 30% compared to control plants [47]. Our results showed that both endophytic strains *B. subtilis* 26D and *B. subtilis* Ttl2 secreted cytokinins and IAA in the culture medium and did not produce ABA (Table 1). Interestingly, the balance between auxin and cytokinin levels is often considered a key regulator of plant organogenesis [65]. We found that *B. subtilis* 26D had a CK/IAA balance of 1.4, and *B. subtilis* Ttl2 had a CK/IAA balance of 0.29 (Table 1). Thus, strains *B. subtilis* 26D and *B. subtilis* Ttl2 had a growth-promoting effect on tomato plants and reduced the damage caused by PVX and PVY, including due to the production of the phytohormones CK and IAA.

### 4.4. Effect of the Endophytic Strains B. subtilis 26D and B. subtilis Ttl2 on the Ribonucleases of Tomato Plants Infected with PVX and PVY

In addition to the production of hormones, PGPM secrete various enzymes, such as proteases, glucanases, nucleases and others. The use of bacterial enzymes can be an alternative and/or additional strategy for protecting plants against viruses. Recent studies have demonstrated that bacterial extracellular ribonuclease effectively inactivates RNA-viruses in plants by cleaving viral RNA and disrupting the formation of a virus coat, most likely due to extracellular ribonuclease [42]. It has been established that *B. cereus* ZH14 produces a new type of extracellular ribonuclease, which is active against TMV [25]. *B. pumilus* ribonuclease directly suppressed the development of potato virus S (PVS) and potato virus M (PVM) infection and also reduced the red clover mottle virus (RCMV) incidence in pea plants [42].

Our results showed that both endophytic strains *B. subtilis* 26D and *B. subtilis* Ttl2 secreted ribonuclease into the culture medium (Table 1) and increased the ribonuclease activity in plants inoculated with viruses (Figure 4). The increase in total ribonuclease activity in plants treated with bacteria and infected with viruses depended on the level of bacterial ribonuclease activity. The 26D strain had a higher ribonuclease activity than the Ttl2 strain, and, accordingly, 26D more strongly activated plant ribonucleases at an early stage of infection, which was accompanied by an increase in the resistance against viral infection. Earlier, the influence of the ribonuclease activity of *Bacillus* spp. on the total ribonuclease activity of potato plants in the field was shown [24]. It should be noted that the ribonuclease activity decreased in the PVY-susceptible VS variety and increased in the PVY-resistant Ural variety (Figure 4). Our results are consistent with the data obtained by other authors, in which a high correlation was found between the ribonuclease activity and the resistance to viruses in different potato varieties [26].

In addition to the general ribonuclease activity, treatment with bacteria induced the transcription of the *PR4* and *PR10* genes, the products of which possess nuclease activity [52,68] in the tomato plants infected with PVX and PVY (Figure 5). Similarly, the level of the *PR4* and *PR10* gene transcripts increased by 4 and 10 times, respectively, in pepper plants treated with *B. amyloliquefaciens* 5B6 and infected with CVM [8]. Moreover, previously, the ribonuclease activity of the PR-10 protein from *Capsicum annuum* was observed towards the Tobacco mosaic virus (TMV) [69]. Thus, the search for endophytic microorganisms that can produce their own ribonucleases and induce plant ribonucleases is a promising strategy for the development of virus control mechanisms in plants.

### 4.5. Effect of the Endophytic Strains B. subtilis 26D and B. subtilis Ttl2 on Triggering the Induced Systemic Resistance of Tomato Plants Infected with PVX and PVY

PGPM, besides having a direct effect on viruses, due to the secretion of enzymes, can have an indirect effect linked to induced systemic resistance (ISR). There are many works on the induction of ISR by PGPM against various viruses [6,8,9,10,12,17,23,27]. However, there are very few works describing the PGPM-mediated induction of ISR against PVX and PVY in potato and tomato plants [10,23]. The defense mechanism triggered by PGPM against viruses depends on the complicated interactions among PGPM, the host plant and the virus, involving mostly the salicylate (SA) signaling pathway and, in some cases, both the SA and jasmonate (JA) pathways [8,10,19,23].

Our results showed that treatment with strains 26D or Ttl2 induced the expression of genes related to the SA-dependent response (*SlPR1b.1* and *SlPR5*) in the tomato plants of both the VS and Ural varieties infected with PVX or PVY (Figure 6). It should be noted that strain 26D more strongly induced the expression of the *SlPR1b.1* and *SlPR5* genes than Ttl2 when the plants were infected with PVX. Previous reports showed that treatment with SA induced resistance to PVX in *N. benthamiana* [70], the accumulation of endogenous SA led to a decrease of the level of PVX in *N. benthamiana* and the PVX levels were higher in NahG plants compared to WT plants [71]. Other authors showed that the SA pretreatment of potato plants of the susceptible cultivar Gala led to an increase in the expression of the *PR-1* and *PR-2* genes and a decrease in the accumulation of PVY in plants [72]. The induction of PR-1 gene expression under the influence of the *B. amyloliquefaciens* strain MBI600 in tomato plants infected with PVY and under the influence of the *B. vallismortis* strain EXTN-1 in potato plants infected with PVX and PVY led to an increase in plant resistance to viruses [10,23]. Several studies have shown that SA plays a key role in plant resistance against both PVX and PVY [71,72,73]. We have demonstrated that 26D and Ttl2 are able to activate the SA defense response against PVX and PVY in tomato plants.

In addition, our results showed that treatment with strain 26D or Ttl2 induced the expression of genes related to the JA-dependent response (*SlPR6* and *SlLOX*) in the tomato plants of both the varieties VS and Ural infected with PVX (Figure 7). These results are consistent with similar data previously published by other authors, in which it was found that treatment with the *B. vallismortis* strain EXTN-1 induced JA-dependent genes in potato plants infected with PVX [23]. Other authors showed that a JA pretreatment enhanced resistance to the cucumber mosaic virus (CMV), tobacco mosaic virus (TMV) and turnip crinkle virus (TCV) in Arabidopsis, tobacco, tomato and hot pepper [74].

We observed a completely different response in plants infected with PVY. The expression of *SlPR6* was increased in resistant cv. Ural and plants of both varieties treated with 26D or Ttl2 (Figure 7). In contrast, the expression of *SlLOX* either decreased or did not change in all treatments, except for plants treated with Ttl2, where the expression of this gene was increased (Figure 7). These results suggest that the expression of *SlPR6* was associated with plant resistance to PVY, while expression of *SlLOX* was not directly associated with plant resistance to PVY. Our results are consistent with the work of other researchers, which showed that the expression of *PR6* was increased in PVY-resistant potato genotypes [75]. It was also shown that the metabolism of oxylipins did not play a significant role in the regulation of PVY accumulation in *N. benthamiana* [76]. In addition, other authors found that treatment with the *B. vallismortis* strain EXTN-1 or treatment with the *B. amyloliquefaciens* strain MBI600 induced JA-dependent genes in potato plants or tomato plants infected with PVY, respectively, but to a lesser degree than genes related to the SA-dependent response [10,23]. Thus, *B. subtilis* 26D and *B. subtilis* Ttl2 synergistically induced genes related to the SA-dependent response and JA-dependent response in tomato plants infected with PVX or PVY.

### 4.6. Effect of the Endophytic Strains B. subtilis 26D and B. subtilis Ttl2 on the Hormonal Balance of Tomato Plants Infected with PVX and PVY

The regulation of the systemic resistance of plants against viruses involves not only SA and JA but also plant hormones associated with growth and abiotic stress, such as cytokinins (CK), auxins and abscisic acid (ABA) [31,34,77]. It has been shown in many works that viral infections result in hormonal disruption in susceptible plants [59,72,77]. PGPM can also affect the hormone content in plants, and they not only affect plant growth and productivity, but also regulate the resistance to abiotic and biotic stresses by changing the hormonal balance of plants. Inoculating wheat plants with the auxin-producing *Paenibacillus illinoisensis* IB 1087 and *Pseudomonas extremaustralis* IB-K13-1A increased root mass and root auxin concentrations [78]. Inoculating lettuce plants with different cytokinin-producing *B. subtilis* strains increased the total cytokinin concentrations and ABA concentrations by 2 times and 2.1 times, respectively [79]. The inoculation of maize plants with the ABA-producing strain of *Azospirillum lipoferum* USA 59b induced an increase in the ABA content in plants and promoted their growth under drought conditions [80]. There is a limited amount of data on the effect of PVX and PVY on the hormonal balance of plants [81,82,83], and there is no published data on the effect of PGPM on the hormonal balance of plants during viral infection.

Our results showed that in tomato plants infected with PVX and PVY, the CK content decreased or did not increase with the development of susceptibility. Only in the Ural variety infected with PVY, the content of CK increased, since it showed average resistance to the virus (Figure 8). Our results are in accordance with the previously published data. It was previously found that in response to infection with PVY or *Potexvirus*, the content of the active forms of CK in plants decreased and the content of the inactive storage forms increased at the early stage of infection [81,82]. Treatment with 26D and Ttl2 led to an increase in the content of CK in both varieties infected with PVX or PVY, which may be associated with an increase in the resistance of these plants to viral infection, due to the activation of the SA-dependent signaling pathway (Figure 8). It is also reported about co-regulation of the CK level with the SA level when tobacco plants are infected with TMV, CMV, PVX and PVY [84]. Generally, high levels of CK can modulate SA signaling and increase the protection against viruses and bacteria through a higher expression of SA-related defense genes [85].

We found that infection with both PVX and PVY increased the IAA content in the shoots of both varieties, excluding the cv. Ural infected with PVY (Figure 8). Our results are in accordance with the previously published data. For example, sugar beet plants infected with beet necrotic yellow vein virus (BNYVV; genus *Benyvirus*, family *Benyviridae*) are characterized by elevated auxin levels [86]. Similarly, rice dwarf virus (RDV; genus *Phytoreovirus*, family *Reoviridae*) triggers auxin biosynthesis in rice [87]. In *Arabidopsis thaliana*, the expression of HC-Pro of the tobacco vein banding mosaic virus (TVBMV; genus *Potyvirus*, family *Potyviridae*), leads to the transcriptional activation of the YUCCA genes and, ultimately, to elevated auxin levels [88]. Moreover, transcriptional changes in the auxin-responsive genes have also been reported for many other plant–virus pathosystems and therefore, seem to be a general response of plants to virus infection [33]. We have shown for the first time that strains 26D and Ttl2 are able to reduce and regulate the level of auxins in infected plants. Ttl2 reduced the auxin content to a lesser degree than 26D, possibly since it produced this hormone itself (Figure 8, Table 1).

Our results showed that PVX and PVY had an opposite effect on the ABA content in tomato plants (Figure 8). The role of ABA in virus infection is not well characterized, and the reported effects of viral infection on ABA concentration vary [31,77]. An increase of the ABA content in virus-infected hosts has been reported for a number of compatible interactions [31,77], and infection by PVY of the resistant potato cultivar Sante did not induce ABA accumulation [83]. In our study, infection by PVY led to an increase in the ABA content in the susceptible cv. VS, while the ABA level decreased in the resistant cv. Ural infected with PVY (Figure 8). These results can be explained by the reports demonstrating the ability of the viral protein PVY HC-Pro to increase ABA levels during the early stages of PVY infection, which was accompanied by a significant virus reproduction without the development of serious symptoms [89].

In contrast, the effect of PVX on the ABA content in tomato plants was the opposite. PVX reduced the ABA content in both varieties, and this was associated with susceptibility. It was shown that an exogenous application of ABA increased virus resistance, and mutations in the ABA pathway increased plant susceptibility to PVX [34,77,90]. ABA-mediated viral resistance has been associated with two mechanisms. Callose deposition at plasmodesmata and the RNA silencing pathway are thought to be the main ABA-dependent antiviral mechanisms [77]. An additional report described the role of the ABA pathway in the development of resistance against PVX via regulation of Argonaute (AGO2) and the mechanism of RNA silencing [34,91,92,93].

In the context of these studies, we obtained intriguing results. We have shown, for the first time, that strains 26D and Ttl2 are able to regulate the ABA levels in plants, depending on the strategy of the virus. Endophytes reduced the ABA content in plants infected with PVY. In other words, bacteria inhibited the multiplication of the virus and did not allow that virus to induce a strong accumulation of ABA [89]. Bacteria inhibited the multiplication of PVX and induced the accumulation of ABA to trigger defense reactions, which led to an increase in the resistance of the endophyte-containing plants. The ambivalent effect of ABA can be explained by the fact that it depends on the infecting strategy of virus, the degree of plant resistance, the stage of disease development and, of course, on the concentration of the hormone. Thus, PGPMs influenced the hormonal balance and, therefore, could successfully regulate the systemic resistance of plants to various viruses.

## 5. Conclusions

We have shown that two endophytic strains *B. subtilis* 26D and *B. subtilis* Ttl2 are able to induce the resistance of tomato plants against two dangerous viruses PVX and PVY in a greenhouse condition. The combination of endophyticity and the production of ribonucleases and phytohormones (CK and IAA) ensured the defense potential of the bacteria. Both strains *B. subtilis* 26D and *B. subtilis* Ttl2 increased the activity of the plant ribonucleases and enhanced the growth and productivity of tomato plants. Treatment with endophytes induced ISR by regulating the hormonal balance in plants and activating the expression of PR genes, which are markers of the SA- and JA-dependent defense responses. For the first time, we have shown that bacteria differentially regulated the levels of CK, IAA and ABA in plants to induce resistance against viruses in plants. The number of endophytic cells, the level of ribonuclease activity and phytohormone production, as well as the balance between the secreted CK/IAA, can play crucial roles in the antiviral activity of the strain. Therefore, microbiologists should pay attention to these indicators when bacterial strains are selected as agents of biocontrol of viruses. Further comprehensive investigations of the role of plant and bacterial hormones in PGPM–plant–virus interactions will contribute to the development of new biotechnological, genetic and breeding approaches to protect agricultural crops.

## Figures and Tables

**Table 1:** CFU content in the internal tissues of potato, the ribonuclease activity in vitro and the content of phytohormones in the culture medium of strains *B. subtilis* 26D and *B. subtilis* Ttl2.

Strain	Endophyticity, CFU * 10^4^/g of Fresh Weight		Ribonuclease Activity, Units/(mL of Liquid Medium per Min)	Phytohormone Level, µg/mL of Culture Medium		
	Shoot	Root		IAA	ABA	Cytokinins **
*B. subtilis* 26D	13.1 ± 1.43 ^a^	0.17 ± 0.03 ^a^	5.23 ± 0.25 ^a^	0.11 ± 0.02 ^a^	0.0 ^a^	0.15 ± 0.007 ^a^
*B. subtilis* Ttl2	7.28 ± 1.22 ^b^	0.99 ± 0.06 ^b^	3.02 ± 0.16 ^b^	0.27 ± 0.01 ^b^	0.0 ^a^	0.08 ± 0.004 ^b^

* CFU, colony-forming units. ** Cytokinins, the sum of zeatin and zeatin riboside. The treatments marked with similar Latin letters in one column do not differ significantly according to the LSD test (n = 6, *p* ≤ 0.05). For the endophytic test, n = 20.

**Table 2:** Effect of the endophytic strains *B. subtilis* 26D and *B. subtilis* Ttl2 on the growth parameters of 2 varieties of tomato plants 2 weeks after infection with PVX and PVY.

Variant of Treatment	Parameter		
	Shoot Height, cm	Fresh Weight of Shoot, g	Dry Weight of Shoot, g
**Cultivar Ural**			
Control	25.9 ± 0.6 ^a^	8.3 ± 0.6 ^a^	0.9 ± 0.04 ^a^
*B. subtilis* 26D	32.2 ± 0.6 ^b^	11.7 ± 0.7 ^b^	1.5 ± 0.06 ^b^
*Bacillus* sp. Ttl2	30.9 ± 0.7 ^b^	11.0 ± 0.6 ^b^	1.5 ± 0.05 ^b^
Potato virus X(PVX)	21.3 ± 0.8 ^c^	4.5 ± 0.3 ^c^	0.4 ± 0.03 ^c^
*B. subtilis* 26D + PVX	25.7 ± 0.9 ^a^	9.3 ± 0.5 ^a^	1.0 ± 0.05 ^a^
*Bacillus* sp. Ttl2 + PVX	25.0 ± 0.5 ^a^	9.6 ± 0.6 ^a^	1.1 ± 0.06 ^a^
Potato virus Y(PVY)	24.6 ± 0.7 ^a^	7.5 ± 0.4 ^a^	0.8 ± 0.05 ^a^
*B. subtilis* 26D + PVY	26.8 ± 0.8 ^a^	9.6 ± 0.5 ^a^	1.1 ± 0.05 ^a^
*Bacillus* sp. Ttl2 + PVY	25.4 ± 0.9 ^a^	8.8 ± 0.6 ^a^	1.0 ± 0.05 ^a^
**Cultivar Volovye Serdtse**			
Control	19.8 ± 0.4 ^a^	6.3 ± 0.4 ^a^	0.7 ± 0.04 ^a^
*B. subtilis* 26D	23.5 ± 0.3 ^b^	6.7 ± 0.3 ^a^	0.8 ± 0.03 ^a^
*Bacillus* sp. Ttl2	22.4 ± 0.8 ^b^	6.9 ± 0.5 ^a^	0.9 ± 0.04 ^a^
Potato virus X(PVX)	14.7 ± 0.9 ^c^	4.9 ± 0.3 ^b^	0.4 ± 0.02 ^b^
*B. subtilis* 26D + PVX	19.5 ± 0.8 ^a^	6.0 ± 0.4 ^a^	0.6 ± 0.03 ^a^
*Bacillus* sp. Ttl2 + PVX	18.8 ± 0.9 ^a^	6.0 ± 0.3 ^a^	0.7 ± 0.04 ^a^
Potato virus Y(PVY)	13.5 ± 0.9 ^c^	4.4 ± 0.2 ^b^	0.4 ± 0.02 ^b^
*B. subtilis* 26D + PVY	19.6 ± 0.9 ^a^	5.5 ± 0.4 ^ab^	0.6 ± 0.04 ^a^
*Bacillus* sp. Ttl2 + PVY	17.1 ± 1.1 ^a^	4.2 ± 0.2 ^b^	0.4 ± 0.02 ^b^

The treatments marked with similar Latin letters in one column in one cultivar do not differ significantly from the control ones according to the LSD test (*p* ≤ 0.05). For the shoot height test, n = 20; for the fresh weight and dry weight test, n = 10.

**Table 3:** Effect of the endophytic strains *B. subtilis* 26D and *B. subtilis* Ttl2 on the fruit yield of 2 varieties of tomato plants 14 weeks after infection with PVX and PVY.

Variant of Treatment	Parameter			
	Number of Fruits, n	Average Fruit Weight, g	Total Weight of Fruits per Bush, g	Yield, % of Control
**Cultivar Ural**				
Control	19.3 ± 0.9 ^a^	103.6 ± 5.6 ^a^	1995.0 ± 32.9 ^a^	100
*B. subtilis* 26D	22.0 ± 1.0 ^a^	143.6 ± 3.8 ^b^	3157.6 ± 66.7 ^b^	158.3 ± 3.3
*Bacillus* sp. Ttl2	20.0 ± 0.6 ^a^	128.7 ± 2.4 ^ab^	2574.0 ± 99.0 ^c^	129.0 ± 5.0
Potato virus X(PVX)	0.0 ± 0.0 ^b^	0.0 ± 0.0 ^c^	0.0 ± 0.0 ^d^	0.0 ± 0.0
*B. subtilis* 26D + PVX	16.3 ± 0.9 ^c^	99.0 ± 2.1 ^a^	1613.3 ± 52.4 ^e^	80.1 ± 2.6
*Bacillus* sp. Ttl2 + PVX	14.0 ± 1.2 ^c^	82.7 ± 4.3 ^d^	1147.3 ± 35.4 ^f^	57.5 ± 1.8
Potato virus Y(PVY)	8.3 ± 0.9 ^d^	82.0 ± 6.1 ^d^	673.0 ± 23.5 ^g^	33.7 ± 1.2
*B. subtilis* 26D + PVY	17.0 ± 0.6 ^c^	124.3 ± 3.0 ^ab^	2111.3 ± 52.6 ^a^	105.8 ± 2.6
*Bacillus* sp. Ttl2 + PVY	12.0 ± 1.2 ^cd^	87.3 ± 3.5 ^d^	1040.0 ± 59.4 ^f^	52.1 ± 3.0
**Cultivar Volovye Serdtse**				
Control	11.7 ± 0.9 ^a^	142.7 ± 4.3 ^a^	1658.0 ± 89.8 ^a^	100
*B. subtilis* 26D	13.3 ± 0.9 ^a^	200.3 ± 5.8 ^b^	2661.0 ± 98.2 ^b^	160.5 ± 5.9
*Bacillus* sp. Ttl2	13.3 ± 1.2 ^a^	162.3 ± 4.3 ^c^	2162.3 ± 190.8 ^c^	130.4 ± 11.5
Potato virus X(PVX)	0.0 ± 0.0 ^b^	0.0 ± 0.0 ^d^	0.0 ± 0.0 ^d^	0.0 ± 0.0
*B. subtilis* 26D + PVX	7.3 ± 1.2 ^c^	71.0 ± 2.1 ^e^	520.7 ± 85.4 ^e^	31.4 ± 5.2
*Bacillus* sp. Ttl2 + PVX	5.0 ± 0.6 ^c^	117.3 ± 9.3 ^f^	576.0 ± 24.0 ^e^	34.7 ± 1.5
Potato virus Y(PVY)	0.0 ± 0.0 ^b^	0.0 ± 0.0 ^d^	0.0 ± 0.0 ^d^	0.0 ± 0.0
*B. subtilis* 26D + PVY	11.0 ± 0.6 ^a^	79.3 ± 2.9 ^e^	874.0 ± 67.1 ^f^	52.7 ± 4.0
*Bacillus* sp. Ttl2 + PVY	6.0 ± 0.6 ^c^	95.0 ± 2.9 ^f^	566.7 ± 37.6 ^e^	34.2 ± 2.3

The treatments marked with similar Latin letters in one column in one cultivar do not differ significantly from the control ones according to the LSD test (n = 10, *p* ≤ 0.05).

## Data Availability

Not applicable.

## Supplementary Materials

The following supporting information can be downloaded at: https://www.mdpi.com/article/10.3390/biom12020288/s1, Figure S1: Presence of potato virus X (PVX) (A,C) and potato virus Y (PVY) (B,D) in the sap of potato plants tested with ELISA (A,B) and Western blot (C,D). (F) Detection of PVX, PVY, potato virus S (PVS), potato virus M (PVM) and potato leaf roll virus (PLRV) by DAS-ELISA in the sap of potato plants of the cultivars Agata and Vineta. Abbreviations: NC, negative control; PC, positive control; St(PVX), potato plants (*Solanum tuberosum*) infected with PVX; St(PVY), potato plants (*Solanum tuberosum*) infected with PVY; M, marker of protein standards, Table S1: The sequences of primers used in qRT-PCR for the transcriptional analysis of *Solanum lycopersicum* L. genes, Table S2: Morphological, physiological and biochemical characteristics of *Bacillus subtilis* Ttl2, Figure S2: Sequence similarity of the *Bacillus* sp. Ttl2 isolate with the *Bacillus*-type strains (MegaLine software), Table S3: Potato virus X (PVX) and potato virus Y (PVY) accumulation in infected and non-infected tomato plants treated with either *B. subtilis* 26D or *B. subtilis* Ttl2, Figure S3: Influence of bacterial treatment on the growth rate of the tomato plant Ural (Ural) (A–C) and Volovye Serdtse (VS) (D–F) cultivars 3–5 weeks after infection with PVX and PVY.
