# Supplementary material for: By Modulating the Hormonal Balance and Ribonuclease Activity of Tomato Plants Bacillus subtilis Induces Defense Response against Potato Virus X and Potato Virus Y
Source: Biomolecules. 2022 Feb 10;12(2):288. doi: 10.3390/biom12020288 (PMC8961569; doi:10.3390/biom12020288)
Supplement: Supplementary file 1 [file biomolecules-12-00288-s001.zip › biomolecules-1557749-supplementary.pdf]

By Modulating the Hormonal Balance and Ribonuclease Activity of Tomato Plants *Bacillus subtilis* Induces Defense Response against Potato Virus X and Potato Virus Y

Svetlana V. Veselova*, Antonina V. Sorokan, Guzel F. Burkhanova, Sergey D. Rumyantsev, Ekaterina A. Cherepanova, Valentin Y. Alekseev, Elena R. Sarvarova, Albina R. Kasimova and Igor V. Maksimov

Institute of Biochemistry and Genetics, Ufa Federal Research Centre, Russian Academy of Sciences, Prospekt Oktyabrya, 71, 450054 Ufa, Russia; fourtyanns@googlemail.com (A.V.S.); guzel_mur@mail.ru (G.F.B.); rumyantsev-serg@mail.ru (S.D.R.); k_cherepanova@mail.ru (E.A.C); valentin-1994@yandex.ru (V.Y.A.); sarvarova_lena@mail.ru (E.R.S.); albinakasimova2014@mail.ru (A.R.K.); igor.mak2011@yandex.ru (I.V.M.)

* Correspondence: veselova75@rambler.ru; Tel.: +7-9173423941

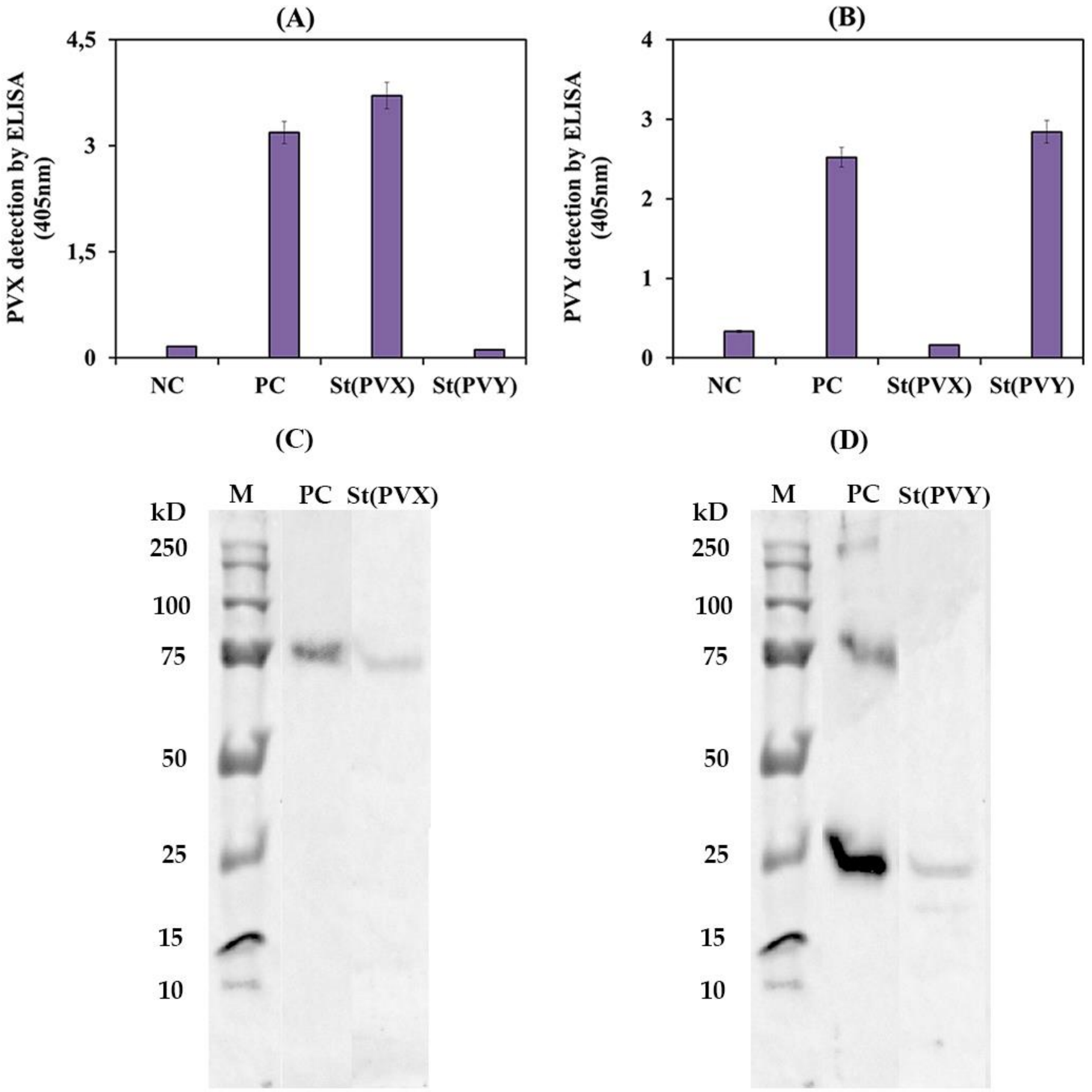

(F)

	Detection of viruses by DAS-ELISA (405 nm)				
**Cultivars**	**PVX**	**PVY**	**PVS**	**PVM**	**PLRV**
Agata	+	-	-	-	-
Vineta	-	+	-	-	-

Figure S1. Presence of potato virus X (PVX) (**A, C**) and potato virus Y (PVY) (**B, D**) in the sap of potato plants tested with ELISA (**A, B**) and Western blot (**C, D**). (**F**) Detection of PVX, PVY, potato virus S (PVS), potato virus M (PVM) and potato leaf roll virus (PLRV) by DAS-ELISA in the sap of potato plants of cultivars Agata and Vineta. Abbreviations: NC – Negative control, PC – Positive control, St(PVX) - potato plants (*Solanum tuberosum*) infected with PVX, St(PVY) - potato plants (*Solanum tuberosum*) infected with PVY, M – marker of protein standards.

Table S1. The sequences of primers used in qRT-PCR for transcriptional analysis of *Solanum lycopersicum* L. genes.

Gene's product	Genes	GenBank Accession number	Sequence (5'-3')	
			Forward Primers	Reverse Primer
Actin	*SlACT*	NM_001308447	CAAGGCCAACAGAGAGAAGAT	GACCGCTAGCATACAGAGAAAG
Lipoxygenase	*SlLOX*	XM_015215079	ACTCATCAGCACCGACATCG	ACTCTCCAGAAAGAACTCCTGC
Proteinase inhibitor, PR6	*SlPR6*	K03291	CCTATTCAAGATGTCCCCGTTC	GGGCAATCCAGAAGATGG
PR1	*SlPR1b.1*	NM_001247385	CGAGAGGCCAAGCTATAACTAC	GCGCCAGACTACTTGAGTATAA
Thaumatin-like protein, PR5	*SlPR5*	NM_001247422	TGTGGTGGAGTCTTGGAATG	AGTTGGGCCGAAAGACATAG
Protein with ribonucleolytic activity, PR4	*SlPR4*	NM_001247154	CAGGAACAGGAACACAAGAAAC	CCTCTGATAGCCCAATCCATTA
Protein with ribonucleolytic activity, PR10	*SlPR10*	KF682292	CTGGAGATGGAGGTTGTGTTT	GGCTTTCCCTTTGCCTACA

Table S2. Morphological, physiological and biochemical characteristics of *Bacillus subtilis* Ttl2

Properties	*Bacillus subtilis* Ttl2
Plant	*Triticum timopheevi*
Organ	leaves
**Morphological characteristics**	
Cell morphology	rod
Spore forming	+
Spore surface	smooth
Colony shape	round
Edges of the colony	irregular
Colony surface	rough matted
Colony color	opaque, beige
**Physiological characteristics**	
Grow at 4°C	-
Grow at 50°C	+
Grow at 60°C	-
Optimum temperature for growth	30°C
pH range for growth	6-8
Optimum pH for growth	7,2
Growth on PDA	+
Growth on LB agar	+
Growth on Bacillus agar, color	green
2,5% NaCl	+
6,5% NaCl	+
Growth under anaerobic condition	-
**Biochemical characteristics**	
Gram's reaction	+
Amylase production	+
Protease production	+
Gelatinase liquefaction	+
Lipase production	-
Catalase production	+
RNase production	+
Urea hydrolysis	-
Vogues Proskauer test	+
NH_3 production	-
Indole production test	+
H_2S production	-
**Growth in presence of different carbon sources**	
Glucose	+
Sucrose	+
Mannitol	+
Lactose	+

Percent Identity (upper triangle); Divergence (lower triangle)

	1	2	3	4	5	6	7	8		
**1**		90.7	91.8	97.2	95.7	99.0	92.8	99.1	**1**	Bacillus amyloliquefaciens MK795392.1
**2**	10.1		89.4	90.6	91.8	91.3	96.1	91.0	**2**	Bacillus cereus KF853584.1
**3**	9.6	12.0		91.9	90.6	91.3	90.9	91.3	**3**	Bacillus endophyticus NR_025122.1
**4**	2.9	10.5	8.6		94.2	97.9	92.2	97.9	**4**	Bacillus licheniformis NR_074923.1
**5**	4.7	9.3	11.0	6.1		95.5	93.3	95.7	**5**	Bacillus pumilus MK184561.1
**6**	1.1	9.6	9.3	2.1	4.7		93.3	99.9	**6**	Bacillus subtilus NR_112116.2
**7**	8.1	4.0	9.5	8.5	8.3	7.6		93.0	**7**	Bacillus thuringiensis KY120371.1
**8**	0.9	9.8	9.1	2.0	4.5	0.1	7.8		**8**	Ttl2
	**1**	**2**	**3**	**4**	**5**	**6**	**7**	**8**		

Figure S2. Sequence similarity of *Bacillus* sp. Ttl2 isolate with *Bacillus* type strains (MegaLine software).

Table S3. Potato virus X (PVX) and potato virus Y (PVY) accumulation in infected and non-infected tomato plants treated with either *B. subtilis* 26D or *B. subtilis* Ttl2.

Variant of treatment	Cultivar Ural				Cultivar Volovye Serdtse			
	Non infected	PVX	Non infected	PVY	Non infected	PVX	Non infected	PVY
**2 Weeks after Infected with Virus, Growth Chamber Conditions**								
Control	–*	3.27 ± 0.25[a]	–	0.46 ± 0.05[a]	–	3.98 ± 0.18[e]	–	1.21 ± 0.03[d]
B. subtilis 26D	–	2.57 ± 0.21[b]	–	0.22 ± 0.01[b]	–	2.17 ± 0.15[b]	–	0.46 ± 0.04[a]
B. subtilis Ttl2	–	2.13 ± 0.16[b]	–	0.59 ± 0.06[a]	–	3.26 ± 0.21[a]	–	0.65 ± 0.07[a]
**10 Weeks after Infected with Virus, Greenhouse Conditions**								
Control	–	2.04 ± 0.17[b]	–	2.85 ± 0.02[c]	–	2.36 ± 0.14[b]	–	2.84 ± 0.19[c]
B. subtilis 26D	–	0.19 ± 0.01[d]	–	0.26 ± 0.01[b]	–	1.77 ± 0.16[c]	–	1.55 ± 0.16[d]
B. subtilis Ttl2	–	1.49 ± 0.15[c]	–	1.35 ± 0.02[d]	–	1.90 ± 0.15[bc]	–	1.72 ± 0.24[d]

* Line indicates absence of viral protein. PVX and PVY were detected by ELISA. Samples were considered positive for presence of PVX or PVY if the absorbance value A405 exceeded three times of a threshold value equal to the mean of the absorbance value of healthy control samples. Latin letters in one column do not differ significantly according to the LSD test (n = 6, $p \le 0.05$).

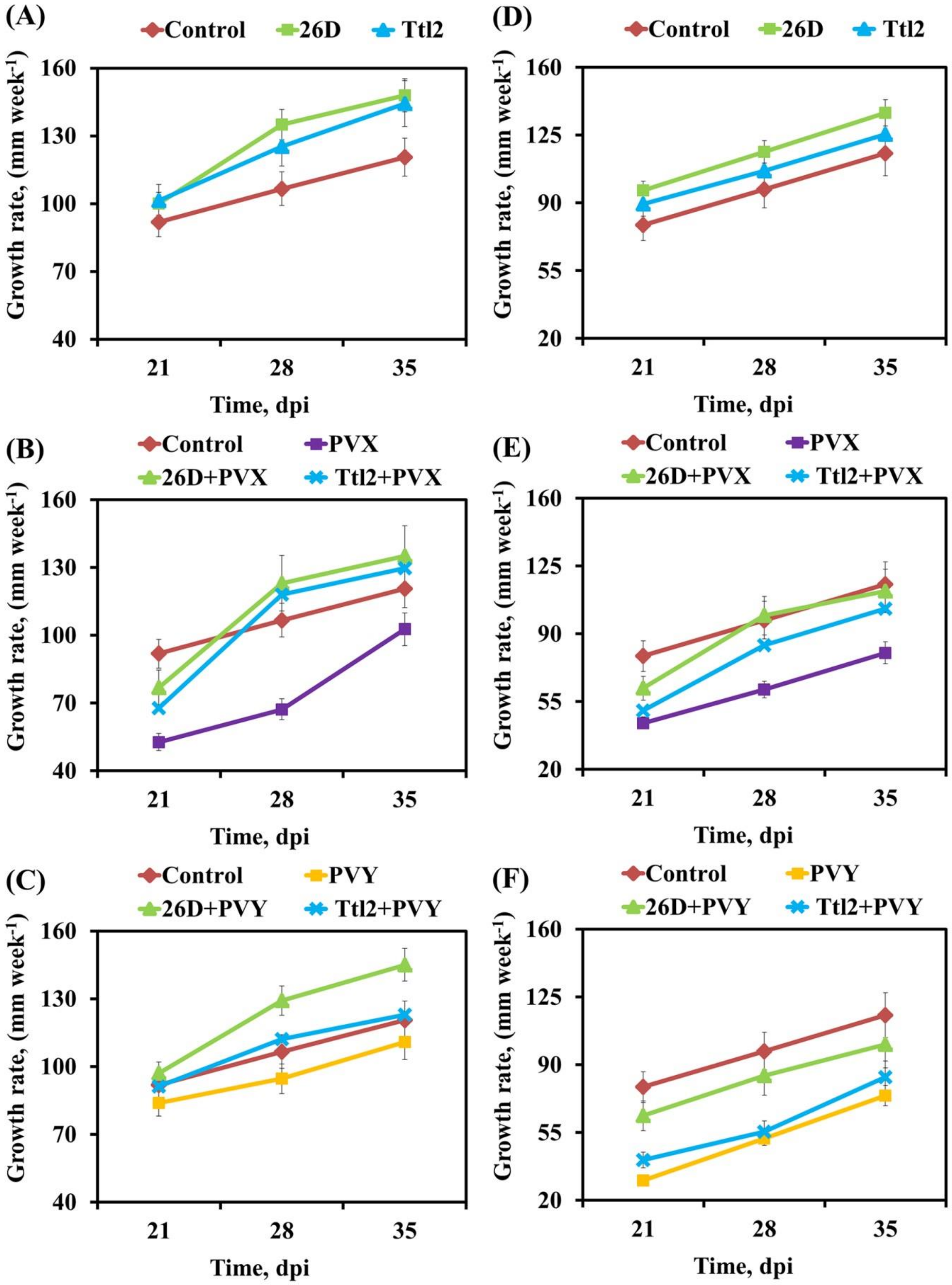

Figure S3. Influence of bacterial treatment on the growth rate of tomato plants Ural (Ural) (A, B, C) and Volovye Serdtse (VS) (D, E, F) cultivar 3 - 5 weeks after infection with PVX and PVY.
